# Decreased carbohydrate sources reduce microbial diversity and taxonomic redundancy in the murine gut

**DOI:** 10.1128/spectrum.03107-25

**Published:** 2026-07-02

**Authors:** Clara Flores, Anna M. Seekatz

**Affiliations:** 1 Department of Biological Sciences, Clemson University 2545, Clemson, South Carolina, USA; University of Nevada Reno, Reno, Nevada, USA

**Keywords:** *Lachnospiraceae*, dietary carbohydrates, taxonomic redundancy, *Akkermansia*, carbohydrate metabolism, gut microbiome

## Abstract

Changes in dietary composition, particularly in carbohydrate diversity, alter the resource environment of the gut microbiome, with downstream effects on community assembly and diversity. However, the extent to which reducing the number of distinct dietary carbohydrates, independent of total carbohydrate intake, shapes microbiota structure remains unclear. This study investigated how reducing the number of dietary carbohydrates, but not total carbohydrate intake, shapes gut microbial diversity in C57BL/6 mice. Over 8 weeks, mice consumed isocaloric diets varying solely in carbohydrate complexity (*n* = 8, 6, or 3 different carbohydrate types) but matched for total carbohydrate content. Using 16S rRNA amplicon sequencing, we found that reducing carbohydrate sources led to significant declines in microbial diversity and taxonomic redundancy among important bacterial groups, such as unclassified Lachnospiraceae, Ruminococcaceae, and Muribaculaceae, despite no immediate changes in host physiology. Concurrently, *Akkermansia* increased under low-complexity diets, independent of the taxonomic redundancy of other species. These changes suggest that loss of diverse sources within a single major nutrient source for microbes (i.e., carbohydrates) narrows microbial niches, which could subsequently disrupt metabolic interactions and functional stability of the gut ecosystem. In the context of modern, industrialized diets often characterized by reduced diversity and structural complexity of carbohydrates, these findings emphasize the importance of diverse nutrient sources in maintaining gut microbial diversity. While short-term host effects were minimal, the microbial shifts observed could presage long-term consequences for gut resilience and disease susceptibility.

**IMPORTANCE:**

Variation in dietary carbohydrate composition shapes the resource environment available to the gut microbiota and can influence microbial community structure and stability. In this study, we show that isocaloric diets with a reduced number of different dietary carbohydrate sources (with total carbohydrate levels constant) significantly altered the structure of gut microbial communities in mice. A reduction in the variety of carbohydrate sources led to decreased microbial diversity and taxonomic redundancy within key microbial groups. These occurred without notable effects on host physiology, although our current study did not focus on long-term host consequences. Our findings suggest that mixtures of structurally distinct carbohydrate sources help sustain the diversity of host-associated microbiomes, in line with ecological theory predicting that reduced resource heterogeneity narrows microbial niches and increases competitive exclusion. These results also shed light into the context of human health, where simplified diets have become increasingly more common.

## INTRODUCTION

The gut microbiome, estimated to harbor microbes that encode up to 100-fold more unique genes than our own genomes, plays a vital role in host physiology, including immune system development (1), colonization resistance against pathogens (2), and the production of necessary metabolites (3). Despite millennia of mutualistic coevolution between hosts and their resident microbes, the gut microbiota remains highly dynamic and responsive to environmental inputs (4–6). Among these, diet is one of the most influential and modifiable determinants of microbial community composition (7). Both short- and long-term dietary patterns have been demonstrated to induce measurable changes in microbial taxa and metabolic outputs (8–11). Accordingly, investigating dietary influences on microbial composition is key to understanding the ecological drivers of gut microbial assembly in host-associated microbiomes.

More broadly, the gut microbiome offers a useful host-associated model to identify how changes in resource diversity impact microbial communities. Anthropogenic modification of environments has often resulted in simplification of resource landscapes, reducing heterogeneity in both natural and host-associated systems (12, 13). Such reductions in resource diversity are frequently associated with declines in taxonomic diversity (14, 15) and, importantly, decreases in taxonomic redundancy—the presence of similar or closely related species. Reduced taxonomic redundancy may, in turn, reflect eroded functional redundancy (16, 17)—the presence of multiple taxa capable of fulfilling the same or similar ecological roles (18), leaving ecosystems vulnerable to functional losses after disturbance (14–17). While prior studies have demonstrated that strongly simplified or altered nutrient inputs can reduce microbial diversity in host-associated and *in vitro* systems (19, 20), less is known about how finer scale variation in dietary resource diversity, within otherwise nutritionally balanced diets, influences microbial community assembly.

Of the macronutrients our diets supply, carbohydrates, both simple and complex, serve as a primary energy source for humans and their associated gut microbes (21). While monosaccharides and disaccharides are rapidly absorbed in the small intestine, complex carbohydrates, particularly dietary fibers and resistant starches, escape host digestion and reach the colon, where they are fermented by diverse bacterial populations. These substrates support a wide range of microbial taxa with specialized enzymatic capabilities, particularly the expression of glycoside hydrolases (GHs), which cleave glycosidic bonds in polysaccharides and oligosaccharides. The diversity and distribution of GHs across microbial genomes reflect taxon-specific metabolic niches (22, 23). Thus, dietary carbohydrate diversity may act as an ecological filter, promoting niche differentiation and taxonomic diversity by supporting a wider range of metabolic capabilities. Prior work has demonstrated that starches derived from different botanical sources (i.e., potato, wheat, and corn) differ in their resistant starch content and molecular architecture (24–26), influencing digestibility and fermentation, thus providing niche variation for saccharolytic taxa (27–29). In contrast, simplified or homogeneous diets may reduce microbial niche availability, increase competition, and lead to reduced taxonomic diversity and potential functional loss (22). These shifts can drive the microbiome into alternate stable states that are less favorable to host health (30, 31). Despite growing interest in the ecological effects of modern, processed diets (32), the specific role of carbohydrate source diversity, independent of total caloric and macronutrient content, in shaping the gut microbiome remains less understood.

In this study, we aimed to investigate how the diversity of dietary carbohydrates influenced taxonomic diversity and redundancy in the murine gut microbiome. We use the term “carbohydrate complexity” to refer to the diversity of carbohydrate sources (i.e., number and type of distinct mono-, di-, and polysaccharides). C57BL/6 mice were fed customized diets that varied in the number of different types of carbohydrates (*n* = 8, 6, or 3 carbohydrate types), but matched in total carbohydrate percentage, for a total of 8 weeks. Using 16S rRNA gene-based sequencing, we compared phylogeny, taxonomic diversity, and taxonomic redundancy within and between mice fed three variable diets. We observed decreased overall taxonomic diversity and redundancy for select taxa in mice fed mid- and low-complexity diets in comparison to mice fed a high-complexity diet. While taxonomic redundancy was reduced in some genera, including Lachnospiraceae and Muribaculaceae, other genera, such as *Akkermansia*, maintained similar levels of taxonomic redundancy, suggesting that the observed shifts in the gut microbiota were not universally distributed across all taxa and overall diversity.

## MATERIALS AND METHODS

### Animals and diets

C57BL/6 mice (6–10 weeks old) were purchased from Jackson Laboratory. Upon arrival, mice were randomly assigned to groups of three per cage (by sex) under equivalent conditions on a 12 h light/dark cycle. All cages, bedding (7084 Teklad Pelleted Paper Bedding), and water were autoclaved, and mice were given γ-irradiated custom diets from Research Diets, New Brunswick, NJ, USA (Table S1; see below). For the first 4 weeks, mice were fed *ad libitum* a high-complexity diet (HCD) (#D22021105). After the 4-week acclimatization period, mice were either maintained on the HCD (*n* = 18) or fed a mid-complexity diet (MCD) (*n* = 18; 50% complex carbohydrates from corn starch and 50% simple carbohydrates; #D22021106) or a low-complexity diet (LCD) (*n* = 18; 87.5% simple carbohydrates; #D22021107). All three diets were tested on equal numbers of male and female mice in two separate experimental replicates (Table S2). Each experimental replicate was conducted independently using the same diet assignments, housing conditions, and sampling schedule, with the second replicate performed to enable more robust evaluation of sex and cage variations as biological variables.

Diets were designed to manipulate the diversity and structural complexity of digestible carbohydrate sources while maintaining isocaloric composition and equivalent total macronutrient content across groups. All diets were based on a standardized purified rodent diet formulation (Research Diets D11112225; Table S2), which served as the baseline for non-carbohydrate components. From this formulation, the composition of digestible carbohydrate sources was systematically modified to generate a gradient in carbohydrate diversity and structural complexity. The HCD contained a mixture of starch sources (corn, wheat, and potato starch) and simple sugars. The MCD contained a reduced diversity of starch sources (corn starch only) and simple sugars, while the LCD contained only simple sugars as digestible carbohydrate sources. Cellulose was included at a constant concentration across all diets as a standardized, non-digestible fiber source to maintain comparable total fiber content between groups and isolate variation in digestible carbohydrate structure as the primary experimental variable. Maltodextrin 10 was included at identical levels in all diets as a processing and pelleting aid required for diet manufacture and was not treated as a variable carbohydrate source in the experimental design. Food intake and energy consumption were measured at the cage level, as food was provided *ad libitum* per cage. Food added throughout the week was weighed at each replenishment, and remaining food was weighed at the end of each week. These values were averaged across mice within each cage (*n* = 3) for downstream analysis.

### Intestinal barrier integrity and gastrointestinal transit time

To assess intestinal barrier function, mice were fasted for 3 h with *ad libitum* access to water prior to oral gavage with 0.2 mL of sterile saline containing 12 mg fluorescein isothiocyanate (FITC)-dextran (4 kDa) (33), 8 mg rhodamine B (70 kDa), and 20 mg creatinine (all reagents from Sigma-Aldrich; #46944, #R9379, and #C4255). After 5 h, blood was collected from anesthetized mice via retro-orbital bleed, and serum was isolated by centrifugation. Fluorescence in serum samples was quantified using a Synergy HT plate reader (BioTek) against freshly prepared standard curves. FITC was measured with excitation/emission wavelengths of 495/525 nm, and rhodamine B was measured at 555/585 nm, respectively.

Gastrointestinal transit time was assessed by oral gavage of 150 µL of a 6% (wt/vol) carmine red dye solution prepared in 0.5% methylcellulose (34). Mice were fasted from both food and water for the duration of the assay. Following gavage, mice were individually housed in sterile 1 mL pipette boxes lined with an absorbent pad. Fecal pellets were examined every 15 min, and transit time was defined as the interval between gavage and the first appearance of red-colored feces. Mice were returned to their home cages immediately after dye detection.

Intestinal permeability and gastrointestinal transit time assays were conducted within the same experimental cohort used for microbiome analyses. Fecal samples for microbiome profiling were collected weekly throughout the experiment for longitudinal analysis. On days when assays were performed, fecal collection occurred prior to the initiation of assay procedures. Cecal samples were collected at terminal euthanasia at the end of the 8-week experimental period, following a 3-day recovery period after completion of physiological testing. This design ensured temporal separation between physiological perturbation and endpoint microbiome sampling and minimized potential confounding effects of assay procedures on microbial community measurements.

### Dual-energy X-ray absorptiometry scan

Animals were weighed prior to euthanasia, after which cecal contents and tissue, ileal contents, and colonic contents and tissue were collected. Carcasses were kept on ice until transport to the dual-energy X-ray absorptiometry (DXA) facility. Upon arrival, animals were allowed to thaw at room temperature and were positioned in a standardized left lateral recumbent orientation for scanning. Whole-body composition was assessed using a Hologic DXA scanner (Discovery A, serial number 87637), operated using the manufacturer’s rat whole-body scan settings. Scans were analyzed using the instrument’s standard analysis software, and the following parameters were recorded: total area, bone mineral content (BMC), bone mineral density, fat mass, lean mass plus BMC, total mass, and percentage of body fat.

### 16S rRNA gene-based sequencing

DNA isolation was done on mouse fecal and cecal content using Qiagen’s MagAttract PowerMicrobiome DNA/RNA kit (#27500-4-EP). The bacterial V4 16S rRNA region was amplified as described previously (35). Briefly, amplicons were generated using a high-fidelity polymerase (Invitrogen, #12-346-086). Libraries were cleaned up and normalized using Invitrogen SequalPrep Plate Normalization Kit (#A1051001). After normalization, sample quality was assessed using the Agilent TapeStation and quantified using KAPA Biosystems quantitative PCR (qPCR) kit. Samples were sequenced on Illumina’s MiSeq platform following the Schloss lab MiSeq Wet Lab standard operating procedure with a 10% PhiX spike according to the manufacturer’s protocol using a final concentration of 4 pM (36). Raw sequence data have been deposited in the Short Read Archive under BioProject PRJNA1308500 (SAMN# 50775611-50776048).

### Absolute abundance of *Akkermansia* species

Genomic DNA (gDNA) was extracted from isolated *Akkermansia muciniphila* strains cultivated from experimental mice using Qiagen’s DNeasy UltraClean Microbial Kit (#12224-250). DNA concentration (ng/µL) was quantified fluorometrically using a Qubit 3 fluorometer (Invitrogen Q33216). Genome copy number µL^−1^ was calculated from Qubit-derived DNA concentrations along with the genome size of the reference *A. muciniphila* strain (2.68 Mbp).

Standard curves were generated using six 10-fold serial dilutions of *A. muciniphila* gDNA using nuclease-free water, spanning a dynamic range of 10^2^–10^7^ gDNA copies µL^−1^. Quantification was performed using primers targeting the 16S rRNA gene of *A. muciniphila* (AM1: 5′-CAGACGTGAAGGTGGGGAC-3′ and AM2: 5′-CCTTGCGGTTGGCTTCAGAT-3′) (37). qPCR reactions were performed on a Bio-Rad CFX Opus Real-Time PCR System (Bio-Rad Laboratories, Hercules, CA, USA) in technical triplicate for all samples and standards, including a no-template control. Each 20 µL reaction contained 1× iQ SYBR Green Supermix (Bio-Rad, #1708880), 300 nM forward primer, 300 nM reverse primer, 1 µL template, and nuclease-free water.

Thermocycling conditions consisted of an initial denaturation step of one cycle of 95°C for 10 min, followed by 35 cycles of 95°C for 15 s, 60°C for 20 s, and 72°C for 30 s each, one cycle of 95°C for 1 min, one cycle of 60°C for 1 min, and a stepwise increase in the temperature from 60°C to 95°C (at 0.5°C per 5 s) to obtain melt curve data. Copy numbers were normalized to the mass of input material used for DNA extraction and are reported as log_10_(16S rRNA gene copies/g). Data were analyzed using the Bio-Rad CFX Maestro Software.

### Microbiome analysis

For 16S rRNA gene-based analysis, sequences were processed using mothur v.1.48.0 (35). Briefly, the SILVA rRNA database project (v132) (38) was used to align reads to the v4 region of the 16S rRNA gene. Sequences were clustered by 100% sequence similarity into amplicon sequence variants (ASVs) and classified using the mothur-adapted version of the Ribosomal DNA Project database (v18) (39). Individual mice were treated as biological samples (*n* = 18 per diet group), while accounting for non-independence due to co-housing by including cage as a random effect in statistical models. Diet group and sex were treated as fixed effects where appropriate, depending on the statistical model. All statistical analyses and data visualization were performed using R (v4.4.0) with dplyr, readaxl, and ggplot2 (40–42). Principal coordinates analysis and permutational multivariate analysis of variance (PERMANOVA) were conducted using the vegan package (43). Data distributions were assessed using the Shapiro-Wilk test for normality and Levene’s test for homogeneity of variance. For comparisons across multiple groups, one-way ANOVA was used when assumptions of normality and homoscedasticity were met; otherwise, the Kruskal-Wallis test was applied. *Post hoc* pairwise comparisons were performed using Tukey’s HSD, Dunn’s test, or Wilcoxon rank-sum tests where appropriate. Differentially abundant ASVs between lower complexity diets and HCD-fed mice were identified using Multivariable Association with Linear Models (MaAsLin2) (44), which allows modeling of covariates and robust testing of associations between microbial taxa and experimental variables while maintaining statistical power. All specific commands and quality parameters are available at https://github.com/SeekatzLab/RoL-FunctionalRedundancy-microbiota.

## RESULTS

### Diets varying in carbohydrate complexity did not impact host physiology

**Fig 1:**
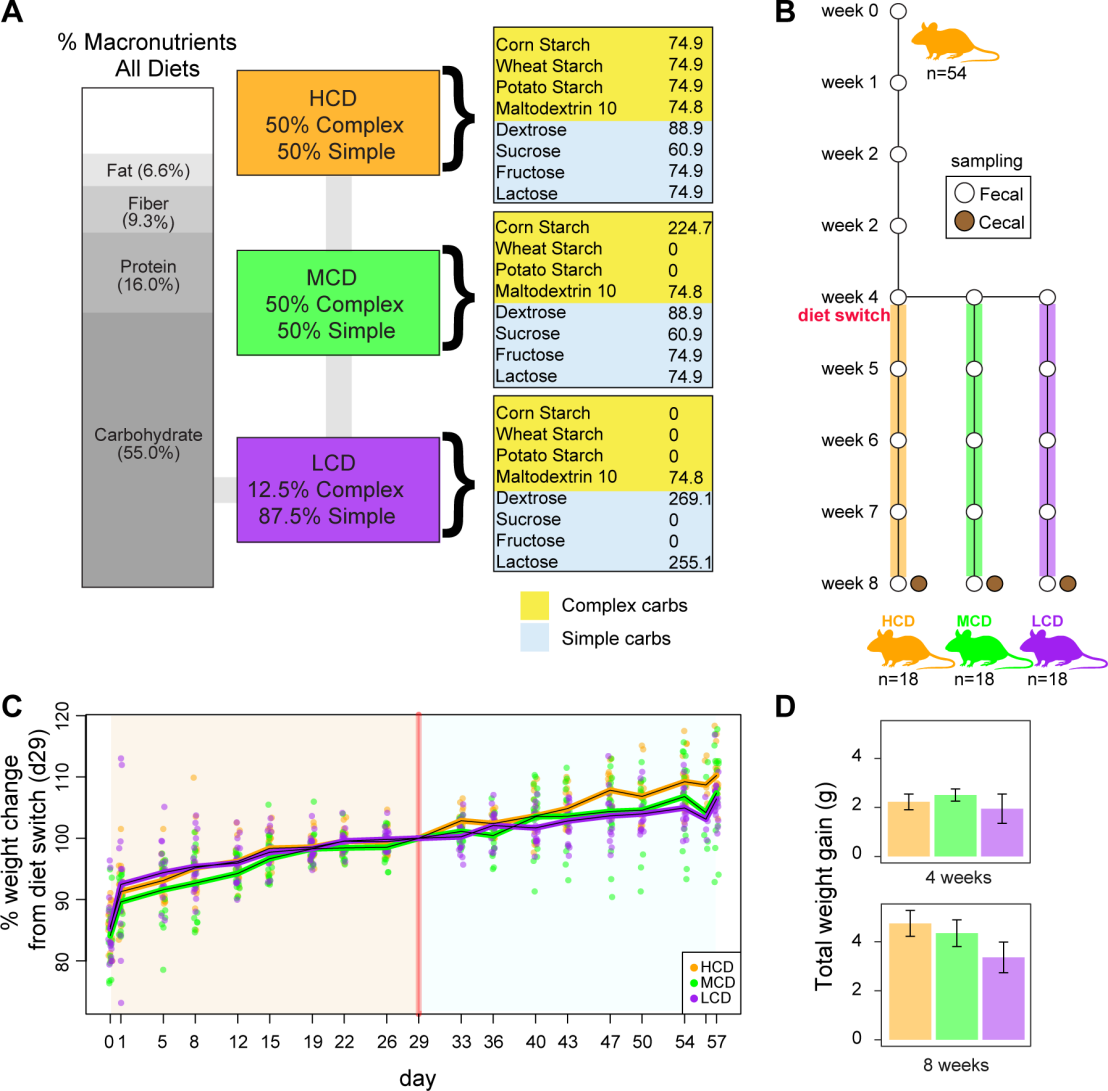
Experimental design and dietary intervention. (**A**) Composition of customized diets designed to measure shifts in gut microbiota taxonomic redundancy in response to carbohydrate complexity. All diets were isocaloric and matched for total macronutrient content (fat, protein, carbohydrate, and fiber), varying only in the composition of carbohydrate sources (proportions of complex vs simple sugars). (**B**) Schematic of experimental design using a mouse model. (**C**) Body weight trajectories of mice over the 8-week study period. Weights were normalized to the day of diet switch (D29). The left panel shows weight change during the initial 4 weeks (pre-diet switch), and the right panel shows weight change during the final 4 weeks (post-diet switch). (**D**) Total weight gain (g) per diet group, averaged over the first 4 weeks and over the full 8-week study period.

To test the effects of dietary carbohydrate complexity on the diversity and taxonomic redundancy of the gut microbiota, we developed three custom diets that exhibited the same percentage of total carbohydrates but differed in composition and number of carbohydrates (Fig. 1A; Table S1). The carbohydrate sources were selected based on their known differences in structural complexity, digestibility, and fermentability, establishing a deliberate gradient from high to low carbohydrate complexity diets (see Materials and Methods). C57BL/6J mice (*n* = 54) were all maintained on the high-complexity diet for 4 weeks, then either continued on the HCD or switched to a MCD or LCD (*n* = 18 per group; Fig. 1B). Mice in all groups steadily gained weight throughout the study (Fig. 1C). Although total weight gain did not differ significantly across diet groups (Fig. 1D), mice fed the LCD exhibited a trend toward a slower rate of weight gain compared to those maintained on HCD (Fig. S1A and B; linear mixed-effects model, *P* = 0.0742). Though female mice consumed fewer weekly and total kilocalories (kcal) than male mice, mice fed lower complexity diets did not consume significantly more total kcal than mice maintained on HCD (Fig. S1C through F). Moreover, a subset of mice (*n* = 18; six per group) scanned for body mass composition metrics, including total fat mass, using dual-energy X-ray absorptiometry did not exhibit differences between treatment groups (Kruskal-Wallis, Fig. S1G through L). Gastrointestinal transit time (Kruskal-Wallis, Fig. S2A) and intestinal barrier integrity (Kruskal-Wallis, Fig. S2B and C) were also not significantly impacted by any of the diet switches.

### Lower complexity diets decreased microbiota diversity and shifted community composition

**Fig 2:**
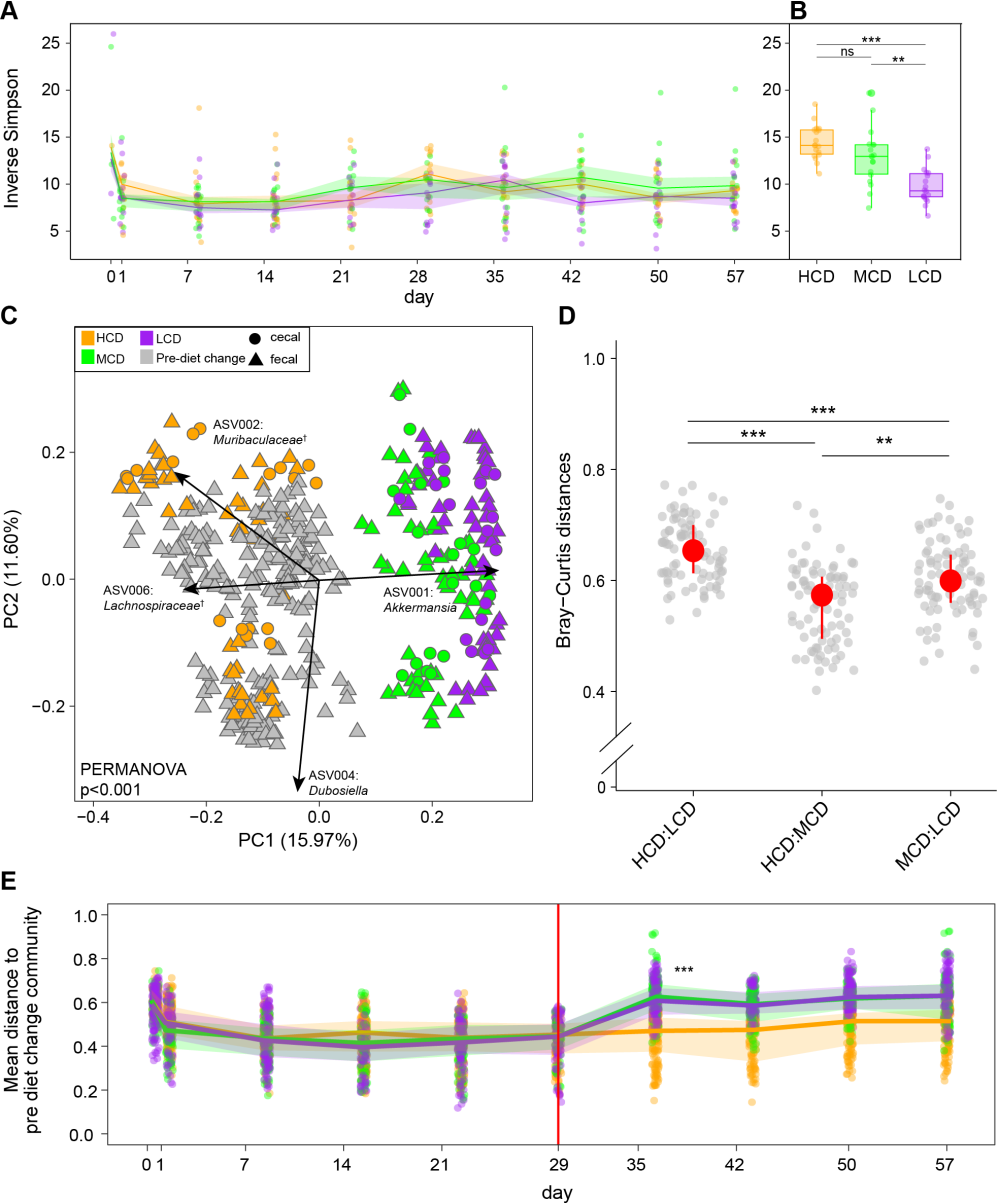
Mid- and low-complexity diets reduce taxonomic diversity. (**A and B**) Inverse Simpson index of (**A**) fecal samples collected throughout the study and (**B**) cecal samples collected at the end of the study. For fecal samples, no significant effect of diet group was detected by linear mixed-effects modeling. (**C**) Principal coordinates analysis of Bray-Curtis dissimilarity based on ASV abundances from fecal and cecal samples (PERMANOVA, *P* < 0.001; † denotes ASVs classified only to the genus level). Arrows represent ASVs contributing to the clustering of cecal communities by diet. (**D**) Pairwise Bray-Curtis dissimilarity between cecal samples by diet group. (**E**) Bray-Curtis dissimilarity over time, calculated as distance from the pre-diet switch (D29). Significant differences between MCD and LCD were compared to HCD controls for all post-diet time points (*P* < 0.001). Statistical significance for panels B and D was assessed using the Kruskal-Wallis with *post hoc* Wilcoxon test (*P < 0.05, **P < 0.01, and ***P < 0.001).

We next sought to identify how the diets impacted the gut microbiota using 16S rRNA gene-based sequencing. Mice fed diets of decreasing complexity (MCD and LCD) demonstrated an overall decrease in diversity in their fecal microbiota following the diet switch and in cecal microbiota at the conclusion of the study, as measured by the inverse Simpson index (Kruskal-Wallis, Fig. 2A and B). Similar patterns were observed across additional alpha diversity metrics analyzed (Fig. S3A through D), with Wilcoxon rank-sum pairwise comparisons indicating significant differences (*P* < 0.05). Changes to the microbiota structure recapitulated the observed changes in diversity, as samples collected from mice fed MCD or LCD clustered away from mice fed HCD or samples collected prior to the diet switch, based on the Bray-Curtis dissimilarity calculated from amplicon sequence variants (Fig. 2C, PERMANOVA, *P* < 0.001 for cecal or fecal comparisons). A biplot based on cecal ASVs overlayed over the observed microbiota structural comparisons revealed four ASVs driving community structure, with ASV001 (*Akkermansia*) driving MCD and LCD group clustering and ASV002 (unclassified Muribaculaceae species), ASV004 (*Dubosiella*), and ASV006 (unclassified Lachnospiraceae species) driving the pre-diet change and HCD community clustering (Fig. 2C). This separation was also evident in endpoint cecal samples, where mice fed HCD were more dissimilar from those fed either MCD or LCD (Fig. 2D). Similarly, while the fecal microbiota of mice maintained on HCD did not differ significantly from their baseline samples, mice fed either MCD or LCD demonstrated a rapid shift in community structure after the diet switch (Kruskal-Wallis, Fig. 2E).

### Lower complexity diets increase *Akkermansia* and decrease ASVs belonging to the family Lachnospiraceae

**Fig 3:**
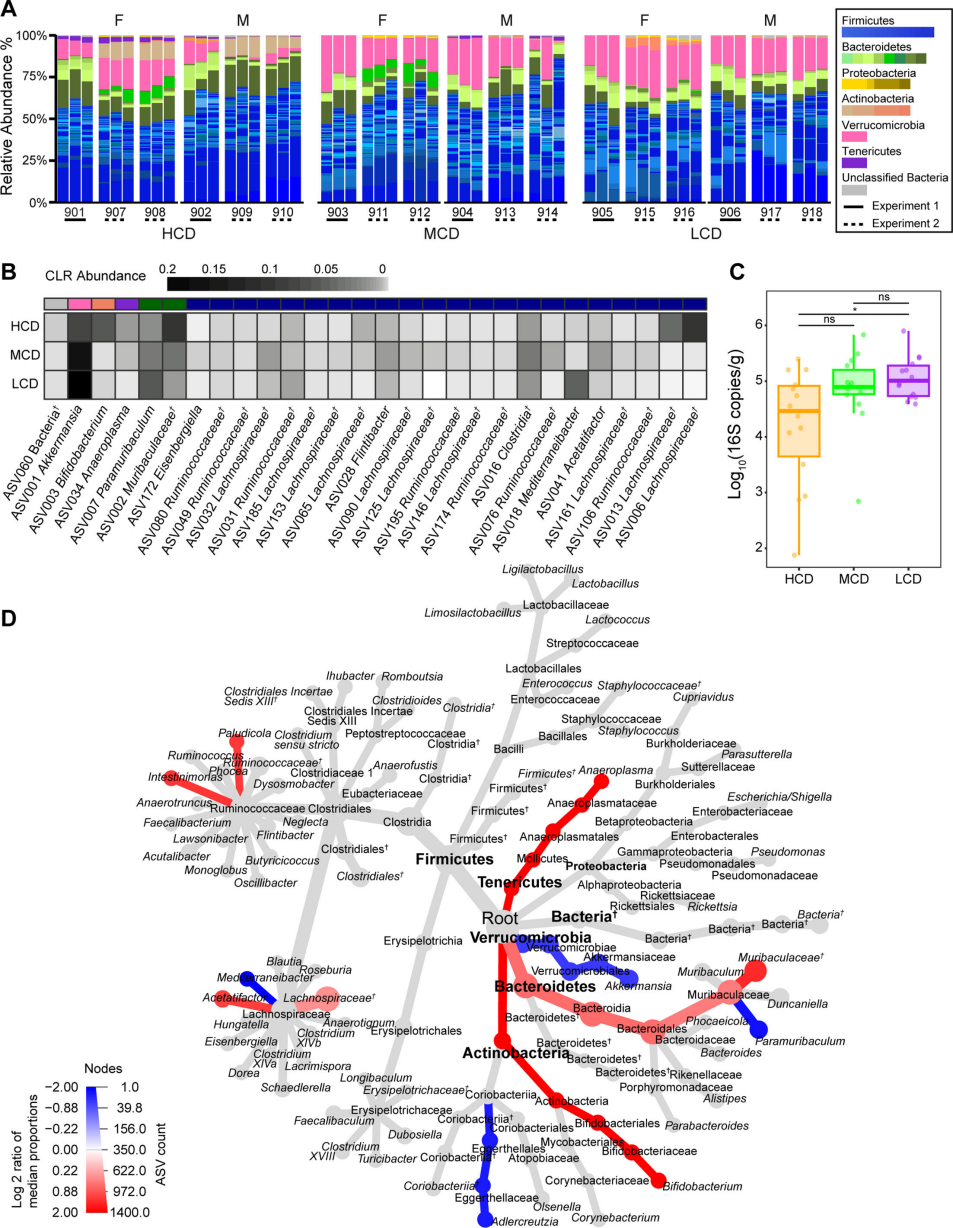
Lower complexity diets increase *Akkermansia* and decrease unclassified Lachnospiraceae. (**A**) Relative abundance of cecal ASVs grouped by diet (HCD, MCD, and LCD), cage ID (901–918), and sex (F, female; M, male). Colors indicate phylum, with genera shown as graded shades within each phylum to preserve phylogenetic structure. (**B**) Mean-centered log-ratio (CLR)-transformed relative abundance of genera identified as significantly different across diet groups compared to HCD-fed mice (MaAsLin2; linear model with Benjamini-Hochberg correction, *q* ≤ 0.1). The right-side color bar corresponds to the phylum-level legend in panel A. (**C**) Absolute abundance of *A. muciniphila* quantified by qPCR and expressed as log10(16S rRNA gene copies/g) across diet groups. Statistical significance was assessed using Kruskal-Wallis with *post hoc* Dunn’s test (*P < 0.05). (**D**) Heat tree comparing LCD- and HCD-fed mice, showing the log_2_ ratio of median taxon proportions. Node size indicates the number of ASVs identified within each taxon detected. Node color reflects the log_2_ fold change between groups, with red colors representing taxa enriched in HCD-fed mice, and blue colors representing taxa enriched in LCD-fed mice. Statistical significance was assessed using the Wilcoxon rank-sum test with FDR correction. † denotes ASVs unclassified at the genus level.

Taxonomic changes in the microbiota mirrored diversity and structural changes due to diet. Across all groups, taxonomic profiling identified genera belonging to six phyla: Actinomycetota (formerly Actinobacteria), Bacillota (Firmicutes), Bacteroidota (Bacteroidetes), Pseudomonadota (Proteobacteria), Mycoplasmatota (Tenericutes), and Verrucomicrobiota (Verrucomicrobia), plus unclassified bacteria. Together, Bacillota and Bacteroidota constituted the majority of the phyla observed across all groups of mice (Fig. 3A), consistent with a healthy murine microbiota. However, within the cecal microbiota, HCD-fed mice exhibited a significantly lower Bacillota/Bacteroidota ratio compared to MCD- and LCD-fed mice (mean ratios: HCD = 2.9; MCD = 4.24; and LCD = 4.23; Wilcoxon, *P* < 0.005) (Fig. S3E). Verrucomicrobiota and Mycoplasmatota were each represented by multiple ASVs representing a single genus, *Akkermansia* and *Anaeroplasma*, respectively.

Significantly higher abundances of *Akkermansia*, mainly represented by ASV001, were observed in MCD- and LCD-fed mice (average 19.1% and 22.4%) compared to HCD mice (9.5%) (Fig. 3A, Wilcoxon, *P* < 0.001). In contrast, *Anaeroplasma*, primarily represented by ASV034, was decreased in mice fed MCD and LCD (0.5% and 0.02%) compared to HCD-fed mice (1.6%). Some experimental variation was observed across cage replicates and/or sex within the groups, most notably by the presence of increased Actinomycetota in cages 907–910 and increased Pseudomonadota in cages 911–912 and 915–916, which represented a second experimental validation (Table S2). In these mice, Actinomycetota, primarily composed of *Bifidobacterium* (ASV003), was significantly decreased in MCD and LCD mice (average 0.05% and 0.25%) compared to HCD mice (9.8%) (Wilcoxon, *P* < 0.001). While only contributing a small percentage of the total relative abundance, *Parasutterella*, belonging to the phylum Pseudomonadota, was increased in these MCD and LCD mice (0.59% and 0.42%) compared to HCD mice (0.23%), the effects of which were enhanced in female compared to male mice (Fig. 3A; Table S3). These findings corroborated the previously observed correlating ASVs in our biplot results (Fig. 2B).

We next assessed differential taxonomic abundance in LCD and MCD groups compared to HCD-fed mice using MaAsLin2, incorporating cage as a random effect and sex as a fixed effect, after filtering ASVs to a minimum relative abundance threshold of 0.001. Across experiments, the relative abundance of *Akkermansia* (ASV001) was significantly increased in mice fed the LCD compared to HCD-fed mice (*q* < 0.05), while mice fed the MCD exhibited a similar but nonsignificant trend (*q* < 0.1); this pattern was particularly evident in male mice, where *Akkermansia* was nearly absent in HCD-fed mice from cage 909 (Fig. 3B). Among the top 28 differentially abundant ASVs, 10 were unclassified Lachnospiraceae, not all of which exhibited the same pattern of differential abundance across the diets. For instance, unclassified Lachnospiraceae ASVs 006, 013, and 065 were increased in HCD-fed mice, while ASV161 and ASV032 were increased in MCD-fed mice. Other taxa significantly decreased in mice fed diets of lower complexity included *Bifidobacterium* (ASV003) and unclassified Muribaculaceae (ASV002) (Fig. 3B). To validate the increase in *Akkermansia* observed in mice fed lower complexity diets, we next quantified absolute abundance using quantitative PCR. Consistent with the differential abundance analysis, the absolute abundance of *Akkermansia* was significantly increased in LCD-fed mice relative to HCD-fed mice (Dunn’s *post hoc* test, *P* < 0.05), while MCD-fed mice exhibited a similar but nonsignificant trend (*P* > 0.1) (Fig. 3C).

Given that the HCD and LCD represented the most contrasting dietary treatments in terms of carbohydrate complexity and diversity and exhibited the largest shifts in community composition and structure, we next conducted a focused comparison between these two groups to further contextualize the observed taxonomic differences. While MaAsLin2 highlighted individual ASVs that were significantly altered between the lower complexity diets and HCD-fed mice, we used a complementary taxon-level approach to explore broader shifts in community structure between HCD- and LCD-fed mice. This analysis revealed distinct patterns of taxon richness and prevalence, with *Anaeroplasma*, *Bifidobacterium*, unclassified Muribaculaceae, and *Intestimonas* more commonly detected in HCD-fed mice, whereas *Akkermansia*, *Paramuribaculum*, and *Adlercreutzia* were more prevalent in LCD-fed mice (Fig. 3D). Within the Lachnospiraceae family, which had one of the highest numbers of assigned ASVs, a majority of the unclassified Lachnospiraceae and *Acetatifactor* ASVs were significantly more prevalent in HCD-fed mice, while *Mediterraneibacter* was more prevalent in LCD-fed mice (Fig. 3D).

### Mice fed lower complexity diets exhibited lower taxonomic redundancy for select taxa

**Fig 4:**
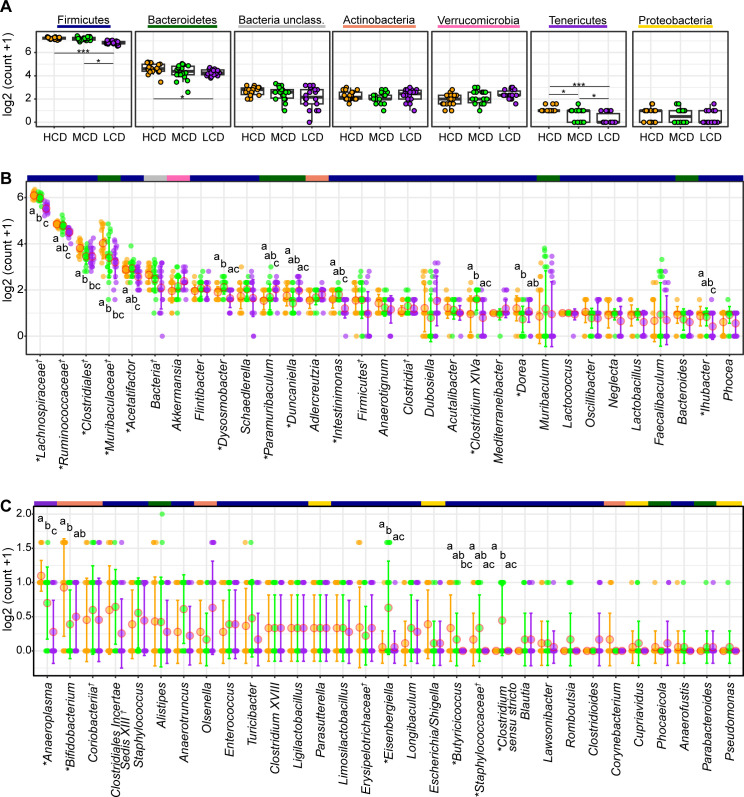
Low-complexity diets resulted in reduced taxonomic redundancy for ASVs observed in Lachnospiraceae but not *Akkermansia*. (**A**) Log_2_ (count + 1) of unique ASV counts per mouse at the phylum level. (**B and C**) Log_2_ (count + 1) of unique ASV counts per genus for the top half (B) and bottom half (C) of genera, ranked by mean ASV count across all samples. Color bars correspond to phylum-level classifications of displayed genera, as displayed in panel A phylum groups. Statistical comparisons for all analyses were performed using Kruskal-Wallis test with *post hoc* Wilcoxon rank-sum tests (*P < 0.05, **P < 0.001, and ***P < 0.0001). * indicates significant differences between at least one pairwise group comparison. † denotes unclassified taxa.

To better understand how the customized diets shaped the gut microbiota, we quantified taxonomic redundancy by determining, at each taxonomic rank (phylum to genus), the number of unique ASVs assigned to each taxon. This allowed us to assess how ASV-level diversity was partitioned across broader taxonomic categories, providing insight into how microbial diversity is organized within different taxonomic levels. At the phylum level, we observed increased Bacillota, Bacteroidota, and Mycoplasmatota unique ASVs in mice fed HCD (Fig. 4A). At the genus level (Fig. 4B), the top three genera with the highest ASV counts (unclassified Lachnospiraceae, unclassified Ruminococcaceae, and unclassified Clostridiales) belonged to the Bacillota phylum. Among these, unclassified Lachnospiraceae showed the highest taxonomic redundancy in HCD-fed mice (average 67.4 unique ASVs), significantly more than in MCD (62.7) and LCD (45.4) groups (Wilcoxon, *P* < 0.05).

Taxonomic redundancy decreased in mice fed LCD within the unclassified Ruminococcaceae*,* unclassified Clostridiales, unclassified Muribaculaceae, and unclassified *Acetatifactor* genera, mirroring the general pattern of decreased relative abundance in these mice (Fig. 4B). While Verrucomicrobiota, represented by a single genus (*Akkermansia*), demonstrated no significant changes across taxonomic levels, Mycoplasmatota, also represented by a single genus (*Anaeroplasma*), had significantly increased redundancy in HCD-fed mice at all taxonomic levels (Fig. S6). Moreover, mice fed LCD had significantly higher ASV counts for *Paramuribaculum* and *Duncaniella*, both of which belong to the phylum Bacteroidota. Specifically, *Duncaniella* was significantly more taxonomically redundant in LCD-fed mice compared to MCD-fed mice (Fig. 4B).

While most genera did not exhibit statistically significant differences in taxonomic redundancy across diet groups, several did—most of which belonged to the phylum Bacillota (Fig. 4B and C; Table S3). Non-significant but consistent trends in taxonomic redundancy were observed from phylum to genus for unclassified bacteria, Pseudomonadota, and Verrucomicrobiota. These patterns are illustrated at the class (Fig. S4), order (Fig. S5), and family (Fig. S6) levels. In contrast, Actinomycetota deviated from the taxonomic redundancy patterns observed at the phylum level. At the family level, Bifidobacteriaceae (Fig. S6) was significantly more taxonomically redundant in HCD-fed mice. Notably, this taxon was absent in the first experiment, highlighting the inherent variability of the microbiota between experimental cohorts.

## DISCUSSION

In this study, we demonstrate that dietary carbohydrate complexity, defined as the diversity of carbohydrate sources, shapes the diversity, composition, and taxonomic redundancy of the gut microbiota in C57BL/6 mice, independent of total carbohydrate content and with minimal measured effects on host physiology. These findings provide evidence that macronutrient composition—not just quantity—can shape gut microbiota structure and function. Reduced carbohydrate complexity was associated with a significant decrease in unclassified Lachnospiraceae species and a corresponding increase in *Akkermansia*, driving shifts in microbial community structure of MCD- and LCD-fed mice away from their pre-diet change and HCD-fed counterparts. Although these and other taxa were differentially abundant in MCD and LCD groups, this did not always correlate with a decrease in taxonomic redundancy, albeit redundancy did decline for select taxa, including Lachnospiraceae, Ruminococcaceae*,* and Muribaculaceae in LCD-fed mice. These findings, combined with existing research linking microbiome composition to inflammation and gastrointestinal disease (45–49), underscore the need to more thoroughly investigate dietary carbohydrate complexity as a modulator of gut microbial ecology.

Despite equivalent macronutrient caloric content across all diets, we observed a clear reduction in diversity and taxonomic redundancy for select taxa as carbohydrate source complexity decreased. These patterns mirror previous findings that microbiota composition and structure are particularly sensitive to sugar composition and fiber content (50–54). Gut microbiota appeared highly responsive to even subtle alterations in substrate complexity, as evidenced by distinct clustering of LCD- and MCD-fed mice from those consuming HCD. In this context, differences in starch composition likely played a role in shaping microbial community structure. The HCD contained corn, wheat, and potato starch, a known source of resistant starch (24, 25, 27), which resists digestion in the small intestine and undergoes fermentation in the colon, effectively extending the pool of fermentable substrates for saccharolytic taxa. In contrast, the MCD included corn starch as its sole starch source, a starch with a relatively lower resistance to digestion (55), while the LCD was devoid of complex starches entirely.

The progressive narrowing of fermentable substrate availability may have imposed niche restrictions, particularly for specialist taxa able to use complex polysaccharides for growth. Resistant starches not only support taxa directly involved in their fermentation, such as *Parabacteroides*, *Ruminococcus*, and *Bifidobacterium*, but also facilitate the expansion of secondary fermenters through cross-feeding interactions (56–58). For instance, increases in taxa not directly involved in resistant starch degradation have been observed in resistant starch-fed animals (59), likely due to their utilization of fermentation byproducts such as oligosaccharides, short-chain fatty acids (SCFAs), or lactate (58). These metabolic interactions help sustain trophic networks, maintain redox balance, and promote microbial ecosystem stability (60, 61). The loss of complex carbohydrates may disrupt these interdependent relationships, limiting metabolic flexibility, constraining the functional potential of the gut microbiota, and ultimately diminishing community resilience.

Notably, the substantial microbial restructuring we observed occurred without changes in body weight, suggesting a degree of short-term host resilience to microbial shifts. This is consistent with Do et al., who observed that high-glucose and high-fructose diets led to significant alterations in gut microbiota composition without accompanying elevations in body weight (62). However, they did report elevated blood glucose and endotoxin levels, indicating early signs of metabolic disruption. In line with our findings, other rodent studies employing higher sugar diets have similarly reported no significant changes in body weight or total caloric intake, despite the presence of physiological or metabolic perturbations (63, 64). While our study did not focus on long-term health consequences to the host, others have observed that a diet rich in simple sugars promotes a pro-inflammatory response via gut microbiota shifts (65), further supporting the notion that microbial alterations may precede overt metabolic dysfunction.

One of the most striking taxonomic changes was the increase in the relative abundance of *Akkermansia* in mice fed lower complexity diets, accompanied by a reduction of Lachnospiraceae species. While our 16S rRNA amplicon results are limited to relative abundance, qPCR measurements concurred with our 16S rRNA amplicon results, demonstrating increased *Akkermansia* in MCD- and LCD-fed mice compared to HCD-fed mice. These results echo findings from Khan et al. (54), where diets supplemented with 10% glucose similarly led to an enrichment of Akkermansiaceae and a decline of Lachnospiraceae. In contrast to fructose- and sucrose-based simple sugar supplementation used in many studies (66, 67), our LCD formulation contained a combination of dextrose and lactose alongside lower levels of more complex carbohydrates. This suggests that the observed *Akkermansia* enrichment may reflect a shift toward increased reliance on host-derived substrates in the relative absence of diverse dietary polysaccharides, a hypothesis supported by prior studies showing increased *Akkermansia* abundance under fiber- or polysaccharide-deprived conditions (68, 69). These findings align with ecological theory predicting that resource heterogeneity promotes niche differentiation and functional redundancy, while resource simplification reduces both, favoring competitive exclusion and the rise of specialist taxa uniquely adapted to limited resources (19, 70).

Though taxonomic redundancy remained largely unchanged across diet groups, MCD- and LCD-fed mice exhibited significantly decreased redundancy for many Bacillota, including Clostridiales, *Dysomobacter*, and Lachnospiraceae, a group broadly associated with health benefits, largely due to their capacity to produce SCFAs (71). Lachnospiraceae, a taxonomically and functionally diverse family composed of both generalists and specialists (72), have been shown to suppress inflammation, and their reduced abundance has been linked to increased susceptibility to colitis (71, 73). Some members may be better adapted to flexible nutrient environments, while others rely more heavily on specific substrates that may include complex carbohydrates whose hydrolysis depends on available GHs (74). In the absence of exogenous carbohydrate sources, as in the MCD and LCD, specialist taxa within Lachnospiraceae may become competitively disadvantaged. In contrast, *Akkermansia muciniphila* is specifically suited to use host mucins as nitrogen and carbon sources (75). This specialization sets *Akkermansia* apart from other generalist microbes, which are not adapted to utilize host-derived glycans (23, 76). The differences in GH repertoire may confer a competitive edge to mucin-degrading Verrucomicrobia under carbohydrate-limited conditions, and future work focused on functional metagenomic profiling and direct quantification of resistant starch fractions or glycan profiles could strengthen conclusions about the functions correlated with our taxonomic changes. Given that our study solely focused on one major nutrient source (carbohydrates), we did not directly measure alternative nutrient utilization pathways, such as amino acids (77), which may contribute to microbial persistence under conditions of reduced carbohydrate diversity.

Over the past several decades, dietary patterns across many populations have shifted markedly from traditional, fiber-rich whole foods toward diets dominated by processed and ultra-processed products (32). These modern diets, while often calorically sufficient and fortified with essential micronutrients, tend to be rich in rapidly digestible carbohydrates derived from a limited set of sources, most notably corn-based sweeteners and starches (78). This reduction in carbohydrate sources parallels a broader trend toward dietary homogenization, with emerging evidence linking such patterns to diminished gut microbial diversity and increased risk of metabolic, inflammatory, and immune-mediated diseases, including obesity, type 2 diabetes, allergies, and inflammatory bowel disease (79). While our study did not focus on a specific health outcome, findings from our mouse model underscore the microbiome’s sensitivity to even modest changes in dietary carbohydrate diversity, independent of total carbohydrate content or caloric load. These alterations, while not accompanied by overt changes in host physiology in the short term, may represent early ecological disruptions that could precede health outcomes dependent on microbiota resilience and functional capacity. Given the growing recognition of the importance of the microbiome’s role in host health, understanding how specific features of diet shape microbial ecosystems will be critical for developing nutritional strategies to maintain or restore gut microbial resilience.

In conclusion, our findings demonstrate that reducing the diversity of dietary carbohydrate sources is sufficient to restructure gut microbial communities and alter patterns of taxonomic redundancy, even in the absence of changes in total carbohydrate intake or overt host physiological effects. However, these conclusions are based on compositional 16S rRNA gene sequencing and therefore do not resolve functional gene content, metabolic activity, or absolute microbial abundance. Additionally, group housing and cage-level measurement of food intake introduce constraints on individual-level exposure resolution. Despite these limitations, the consistency of diet-associated effects across cohorts and analytical frameworks supports a robust ecological signal linking carbohydrate resource diversity to gut microbial community structure. Future work integrating multi-omics approaches will be essential to resolve functional implications of these compositional shifts and determine their relevance to host physiology over longer timescales.

## Supplementary Material

Reviewer comments

## SUPPLEMENTAL MATERIAL

Supplemental figures spectrum.03107-25-s0001.pdf Fig. S1 to S6.

Table S1 spectrum.03107-25-s0002.tiff Dietary components.

Table S2 spectrum.03107-25-s0003.xlsx Mouse metadata.

Table S3 spectrum.03107-25-s0004.xlsx Taxonomic redundancy—significantly different genera.

OPEN PEER REVIEW reviewer-comments.pdf An accounting of the reviewer comments and feedback.

ASM does not own the copyrights to Supplemental Material that may be linked to, or accessed through, an article. The authors have granted ASM a non-exclusive, world-wide license to publish the Supplemental Material files. Please contact the corresponding author directly for reuse.
