# Supplementary material for: Decreased carbohydrate sources reduce microbial diversity and taxonomic redundancy in the murine gut
Source: Microbiol Spectr. 2026 Jul 2;14(8):e03107-25. doi: 10.1128/spectrum.03107-25 (PMC13436403; doi:10.1128/spectrum.03107-25)
Supplement: Supplementary file 1 [file reviewer-comments.pdf]

AMERICAN SOCIETY FOR MICROBIOLOGY | Microbiology Spectrum

Decreased carbohydrate sources reduce microbial diversity and taxonomic redundancy in the murine gut

Clara Flores and Anna Seekatz

Corresponding Author(s): Anna Seekatz, Clemson University

Review Timeline:

Submission Date:	September 29, 2025
Editorial Decision:	February 12, 2026
Revision Received:	May 8, 2026
Accepted:	May 14, 2026

Editor: Steven Frese

Reviewer(s): The reviewers have opted to remain anonymous.

Transaction Report:

(Note: With the exception of the correction of typographical or spelling errors that could be a source of ambiguity, letters and reports are not edited. The original formatting of letters and referee reports may not be reflected in this compilation.)

DOI: https://doi.org/10.1128/spectrum.03107-25

Re: Spectrum03107-25 (**Decreased carbohydrate sources reduce microbial diversity and taxonomic redundancy in the murine gut**)

Dear Dr. Anna Maria Seekatz:

I apologize that this has taken so long, there were some challenges in getting reviews back from reviewers.

Thank you for the privilege of reviewing your work. Below you will find my comments, instructions from the Spectrum editorial office, and the reviewer comments.

Please return the manuscript within 60 days; if you cannot complete the modification within this time period, please contact me. If you do not wish to modify the manuscript and prefer to submit it to another journal, notify me immediately so that the manuscript may be formally withdrawn from consideration by Spectrum.

Revision Guidelines
To submit your modified manuscript, log into the submission site at https://spectrum.msubmit.net/cgi-bin/main.plex. Go to Author Tasks and click the appropriate manuscript title to begin. The information you entered when you first submitted the paper will be displayed; update this as necessary. Note the following requirements:

• Upload point-by-point responses to the issues raised by the reviewers in a file named "Response to Reviewers," NOT in your cover letter.
• Upload a compare copy of the manuscript (without figures) as a "Marked-Up Manuscript" file.
• Upload a clean .DOC/.DOCX version of the revised manuscript and remove the previous version.
• Each figure must be uploaded as a separate, editable, high-resolution file (TIFF or EPS preferred), and any multipanel figures must be assembled into one file.
• Any supplemental material intended for posting by ASM should be uploaded with their legends separate from the main manuscript. You can combine all supplemental material into one file (preferred) or split it into a maximum of 10 files with all associated legends included.

For complete guidelines on revision requirements, see our Submission and Review Process webpage. Submission of a paper that does not conform to guidelines may delay acceptance of your manuscript.

Data availability: ASM policy requires that data be available to the public upon online posting of the article, so please verify all links to sequence records, if present, and make sure that each number retrieves the full record of the data. If a new accession number is not linked or a link is broken, provide Spectrum production staff with the correct URL for the record. If the accession numbers for new data are not publicly accessible before the expected online posting of the article, publication may be delayed; please contact production staff (Spectrum@asmusa.org) immediately with the expected release date.

Publication Fees: For information on publication fees and which article types are subject to charges, visit our website. If your manuscript is accepted for publication and any fees apply, you will be contacted separately about payment during the production process; please follow the instructions in that e-mail. Arrangements for payment must be made before your article is published.

ASM Membership: Corresponding authors may join or renew ASM membership to obtain discounts on publication fees. Need to upgrade your membership level? Please contact Customer Service at Service@asmusa.org.

The ASM Journals program strives for constant improvement in our submission and publication process. Please tell us how we can improve your experience by taking this quick Author Survey.

Thank you for submitting your paper to Spectrum.

Sincerely,
Steven Frese
Editor
Microbiology Spectrum

Reviewer #2 (Comments for the Author):

The manuscript by Flores & Seekatz investigated the effect of carbohydrate complexity on the gut microbiota in a murine model. Using conventional mice and 16S rRNA gene sequencing, the researchers reported that low-complexity carbohydrate diets containing simple sugars resulted in significantly lower bacterial diversity, altered microbiota composition, and higher Akkermansia abundance compared with high-complexity diets. The study provides an important scientific foundation for

understanding diet-microbiome interactions. While the manuscript is logical and meaningful, several methodological aspects require clarification or improvement to support the impact of the research to meet the publication criteria. The concerns are described in more detail below:

Major concerns

- The study's central limitation is its reliance on 16S sequencing, which provides bacterial compositional changes but offers no direct evidence of microbial functional outcomes. The following analyses will significantly enhance the impact of the work: (1) Metagenomic sequencing to identify shifts in functional gene families and metabolic pathways (e.g., GHs); and/or (2) Metabolomic analysis (e.g., by LC-MS or NMR) to quantify key microbial metabolites (e.g., SCFAs). (3) As a minimum alternative, PICRUSt2 could be used for functional prediction from the existing 16S data.

- The effect of diet on total bacterial load remains unmeasured. A qPCR analysis of total bacteria (targeting the 16S gene) is recommended to determine if the observed shifts in relative abundance reflect true changes in bacterial density.

- Line 203, the authors claimed that "the rate of weight gain for mice fed LCD was significantly slower compared to those maintained on HCD (linear mixed-effects model, $p < 0.0001$)." However, the current Figure 1D does not fully support this conclusion. A spaghetti plot connecting the weight of the same mouse from Week 4 to Week 8 may be helpful to visually demonstrate the rate of weight gain.

- Line 224: Could the authors further explain the rationale for using AMOVA? Microbial beta-diversity is widely analyzed by PERMANOVA/Adonis (https://pmc.ncbi.nlm.nih.gov/articles/PMC5695134), with both P-value and R-squared value reported in the main text and figures.

- Line 264: The choice of MaAsLin2 for differential abundance analysis should be justified. A study (https://www.nature.com/articles/s41467-022-28034-z#Abs1) showed that ALDEx2 and ANCOM-II produce the most consistent results across studies and agree best with the intersection of results from different approaches.

- Figure 3A: (1) The plot suggests a potential cage effect. The sex and cage effect should be tested and included as covariates in the statistical analysis of the microbiome data. (2) The use of multiple shades within a phylum is distracting. A single, distinct color per phylum is recommended for clarity.

- Figure 3B: The CLR (centered log-ratio) abundance is recommended to represent relative abundance in visualizing and analyzing compositional data (https://pmc.ncbi.nlm.nih.gov/articles/PMC5695134).

- Figure 3C: The figure and its legend require a more precise description. Based on the legend title, my interpretation is that the node size shows ASV count within each taxa, while the color (black vs brown) represents the log2 fold-change to indicate the taxa is more or less abundant. Additionally, a standard diverging color scheme (e.g., blue for decrease, red for increase) would be more intuitive than black/brown.

Minor concerns

- To validate the key finding on Akkermansia, qPCR analysis targeting this species is recommended. This is necessary to confirm whether its increased relative abundance represents an absolute increase in its population or is a compositional effect driven by the decrease of other taxa.

- Line 290: Since the author used the number of unique ASVs in the redundancy analysis, it will be more reasonable and consistent to use the index "observed ASVs" to represent alpha diversity and move the current Figure 2A to the supplement.

Figure 1B: The mouse schematic is inaccurate and misleading, as the tail is incorrectly depicted and resembles that of a squirrel.

- Figure 2 A, B, C, E: P values need to be added to clearly indicate the significance of the comparisons shown.

In summary, this study reveals diet-induced alterations in bacterial diversity, taxonomic redundancy, and specific species under distinct carbohydrate complexity. However, the conclusions are currently limited to taxonomic observations. It is recommended to address the major points concerning functional validation, statistical rigor, and data presentation. Incorporating these changes will significantly enhance the manuscript's contribution to the field.

Reviewer #3 (Comments for the Author):

The study from Flores and Seekatz investigates how gut microbial community in murine gut reacts to different carbohydrate sources that differ in complexity of their chemical structure. The notion appears to arise from broader claims suggesting that human-driven environmental changes lead to evolutionary simplification of ecosystems. Although such patterns were shown in

other ecological systems, the application of this concept to human evolution and dietary transitions is far less straightforward. Urbanization, in the context of diet, does not really produce simplified dietary environment, but rather complex. The availability of variety of foods, transportation, and having access to different food types contribute to the complexity. Supporting this, the study from DeFillipo et al 2010 showed that urban European children exhibited more complex gut microbial composition than children living in in Burkina Faso, who consume traditional and fiber-rich diets https://pubmed.ncbi.nlm.nih.gov/20679230. Given this, the way intro, abstract, and importance sections were currently written makes it very hard to grasp the hypothesis and objectives of the study. The ultimate goals are about the effects of nutrient complexity and availability on microbial diversity. The narrative sets the readers up to expect an investigation grounded in evolutionary ecology rather than a mechanistic test of how nutrient availability and CHO structural complexity shape microbial community. Importantly, this focus does not emerge until line 80. To improve clarity, the authors should reframe the introduction from the outset, reflecting the true drivers of the experimental design and results.

In addition to concerns about narrative, the study design is questionable and raises concerns over the interpretation of the results. Particularly, Figure 1A is very confusing. Maltodextrin 10 is not labeled as complex carbs in the figure, which is incorrect as maltodextrin is a polysaccharide. In addition, LCD contains 12.5% complex CHO but there is no yellow label. This seems like an error and adds to the confusion on what authors consider complex CHO. Furthermore, labeling fiber differently than CHO is scientifically inaccurate. The figure seems to treat cellulose as the only fiber source, which is reductive and inaccurate. Even though cellulose is not nutritionally valuable from host perspective, some gut microbes utilize cellulose https://pmc.ncbi.nlm.nih.gov/articles/PMC7615765/. Additionally, certain carbohydrate sources labeled here as complex carbs such as corn starch can function as resistant starch, which is operationally a form of dietary fiber. Without correcting these foundational ambiguities, the mechanistic conclusions about carbohydrate complexity and microbial diversity remain difficult to evaluate.

In addition to the issues with diet categorization, animal study design raises major concerns, particularly regarding microbial variability across animals and cages. The mice used in the study ranged from 6 to 10 weeks of age, which represents a substantial developmental window from a microbiome perspective. Is there preliminary data showing the gut microbiome is stable across time without any perturbations? This age range may also explain the sex and cage variation mentioned in lines 252-255. If animals entered the study with differing microbiome maturity levels, these pre existing differences would likely amplify cage effects. Another inconsistency is found in the labeling and interpretation of Experiment 2nd in Figure 3, where the text indicates the variation observed in these cages prompted authors to conduct the 2nd validation experiments. Furthermore, in the microbiome analysis, the authors combine the fecal sample 16S data with the cecal sample which is represented in Figure 2C. Compositionally and naturally, fecal samples which are derived from distal colon and cecal samples are distinct. Naturally, cecal samples will also contain microorganisms that are also attached to the mucus whereas fecal samples contain flushed out microorganisms, aka ileal content. Therefore, combining these data in the same ordination or diversity analysis without accounting for inherent biological variance introduces confounding interpretation. Another drawback is that the ambiguity on what counts as technical replicates and what counts as biological replicates. A fecal sample collected from mice housed in the same cage should be treated as technical replicates, not biological replicates because mouse co-housed in the same cage will share a common microbial environment and should naturally converge toward similar microbial profiles. Thus, a cage constitutes an n=1 for microbiome analysis unless individually housed. Without explicit sample size reporting, the validity of statistical comparisons cannot be assessed. Additionally, it is unclear whether the cecal samples included in Figure 2 and subsequent analyses were collected immediately after the gut transit/permeability assays or whether they derive from a separate cohort of mice. If these samples were obtained following transit or permeability measurements, the physiological perturbations imposed by those assays could independently alter the cecal microbiota. This ambiguity significantly undermine confidence in the microbiome analyses and limit the reliability of the conclusions drawn.

Another concern relates to the caloric intake analysis, which directly impacts the interpretation of the dietary intervention. The authors report that female mice consumed fewer calories overall. This finding challenges the assumption that carbohydrate intake remained equal across groups, because actual intake, not the theoretical diet composition, determines nutrient exposure. If female mice consumed fewer total calories, then their absolute carbohydrate intake was not held constant, which could independently influence microbial composition and physiological outcomes. This discrepancy could also contribute to variability observed across cohorts and sex. These were never addressed as limitations in the discussion. Moreover, the validation of this effect is insufficient, as the authors do not correlate caloric intake with microbial outcomes or examine whether intake differences could account for the reported diversity shifts. If cage is the true biological unit for dietary exposure, then using individual mice as independent microbiome replicates creates a mismatch between the exposure variable (intake) and the microbiome outcome.

Minor revisions:
Line 242, cited wrong figure.
Figure 2 indicates the authors performed AMOVA, do they mean PERMANOVA? Because in the M&M section, they mention that they performed PERMANOVA.
Figure 3A cages 907, 9098, 911 and 912 contain a green color that is not depicted in the legend.
From Figure 3A, it is very hard to understand the genus levels as authors claim in lines 256-261 because the figure legend only indicates phyla level. The authors should cite Table S2 at the same time.
Lines 297-299, percentages should be given.
Line 193 in the Results section, the "host health" is an overinterpretation of the results although the measurements include

calories, body weight, and fat mass there is no metabolic health measurements in serum.
There is no clear conclusion paragraph.
Line 350, the discussion text was submitted without proof reading, still include draft version. The authors' note to themselves: (ref)
Lines 375-376, the Akkermansia abundance in LCD is repeated twice.
Line 334 needs to mention unclassified.
Line 182 data visualization is a repeat in the same sentence.

The study from Flores and Seekatz investigates how gut microbial community in murine gut reacts to different carbohydrate sources that differ in complexity of their chemical structure. The notion appears to arise from broader claims suggesting that human-driven environmental changes lead to evolutionary simplification of ecosystems. Although such patterns were shown in other ecological systems, the application of this concept to human evolution and dietary transitions is far less straightforward. Urbanization, in the context of diet, does not really produce simplified dietary environment, but rather complex. The availability of variety of foods, transportation, and having access to different food types contribute to the complexity. Supporting this, the study from DeFillipo et al 2010 showed that urban European children exhibited more complex gut microbial composition than children living in in Burkina Faso, who consume traditional and fiber-rich diets https://pubmed.ncbi.nlm.nih.gov/20679230. Given this, the way intro, abstract, and importance sections were currently written makes it very hard to grasp the hypothesis and objectives of the study. The ultimate goals are about the effects of nutrient complexity and availability on microbial diversity. The narrative sets the readers up to expect an investigation grounded in evolutionary ecology rather than a mechanistic test of how nutrient availability and CHO structural complexity shape microbial community. Importantly, this focus does not emerge until line 80. To improve clarity, the authors should reframe the introduction from the outset, reflecting the true drivers of the experimental design and results.

In addition to concerns about narrative, the study design is questionable and raises concerns over the interpretation of the results. Particularly, Figure 1A is very confusing. Maltodextrin 10 is not labeled as complex carbs in the figure, which is incorrect as maltodextrin is a polysaccharide. In addition, LCD contains 12.5% complex CHO but there is no yellow label. This seems like an error and adds to the confusion on what authors consider complex CHO. Furthermore, labeling fiber differently than CHO is scientifically inaccurate. The figure seems to treat cellulose as the only fiber source, which is reductive and inaccurate. Even though cellulose is not nutritionally valuable from host perspective, some gut microbes utilize cellulose https://pmc.ncbi.nlm.nih.gov/articles/PMC7615765/. Additionally, certain carbohydrate sources labeled here as complex carbs such as corn starch can function as resistant starch, which is operationally a form of dietary fiber. Without correcting these foundational ambiguities, the mechanistic conclusions about carbohydrate complexity and microbial diversity remain difficult to evaluate.

In addition to the issues with diet categorization, animal study design raises major concerns, particularly regarding microbial variability across animals and cages. The

mice used in the study ranged from 6 to 10 weeks of age, which represents a substantial developmental window from a microbiome perspective. Is there preliminary data showing the gut microbiome is stable across time without any perturbations? This age range may also explain the sex and cage variation mentioned in lines 252-255. If animals entered the study with differing microbiome maturity levels, these pre-existing differences would likely amplify cage effects. Another inconsistency is found in the labeling and interpretation of Experiment 2nd in Figure 3, where the text indicates the variation observed in these cages prompted authors to conduct the 2nd validation experiments.

Furthermore, in the microbiome analysis, the authors combine the fecal sample 16S data with the cecal sample which is represented in Figure 2C. Compositionally and naturally, fecal samples which are derived from distal colon and cecal samples are distinct. Naturally, cecal samples will also contain microorganisms that are also attached to the mucus whereas fecal samples contain flushed out microorganisms, aka ileal content. Therefore, combining these data in the same ordination or diversity analysis without accounting for inherent biological variance introduces confounding interpretation. Another drawback is that the ambiguity on what counts as technical replicates and what counts as biological replicates. A fecal sample collected from mice housed in the same cage should be treated as technical replicates, not biological replicates because mouse co-housed in the same cage will share a common microbial environment and should naturally converge toward similar microbial profiles. Thus, a cage constitutes an n=1 for microbiome analysis unless individually housed. Without explicit sample size reporting, the validity of statistical comparisons cannot be assessed. Additionally, it is unclear whether the cecal samples included in Figure 2 and subsequent analyses were collected immediately after the gut transit/permeability assays or whether they derive from a separate cohort of mice. If these samples were obtained following transit or permeability measurements, the physiological perturbations imposed by those assays could independently alter the cecal microbiota. This ambiguity significantly undermine confidence in the microbiome analyses and limit the reliability of the conclusions drawn.

Another concern relates to the caloric intake analysis, which directly impacts the interpretation of the dietary intervention. The authors report that female mice consumed fewer calories overall. This finding challenges the assumption that carbohydrate intake remained equal across groups, because actual intake, not the theoretical diet composition, determines nutrient exposure. If female mice consumed fewer total calories, then their absolute carbohydrate intake was not held constant, which could independently influence microbial composition and physiological outcomes. This discrepancy could also contribute to variability observed across cohorts and sex. These

were never addressed as limitations in the discussion. Moreover, the validation of this effect is insufficient, as the authors do not correlate caloric intake with microbial outcomes or examine whether intake differences could account for the reported diversity shifts. If cage is the true biological unit for dietary exposure, then using individual mice as independent microbiome replicates creates a mismatch between the exposure variable (intake) and the microbiome outcome.

Minor revisions:

Line 242, cited wrong figure.

Figure 2 indicates the authors performed AMOVA, do they mean PERMANOVA? Because in the M&M section, they mention that they performed PERMANOVA.

Figure 3A cages 907, 9098, 911 and 912 contain a green color that is not depicted in the legend.

From Figure 3A, it is very hard to understand the genus levels as authors claim in lines 256-261 because the figure legend only indicates phyla level. The authors should cite Table S2 at the same time.

Lines 297-299, percentages should be given.

Line 193 in the Results section, the “host health” is an overinterpretation of the results although the measurements include calories, body weight, and fat mass there is no metabolic health measurements in serum.

There is no clear conclusion paragraph.

Line 350, the discussion text was submitted without proof reading, still include draft version. The authors’ note to themselves: (ref)

Lines 375-376, the Akkermansia abundance in LCD is repeated twice.

Line 334 needs to mention unclassified.

Line 182 data visualization is a repeat in the same sentence.

We thank reviewers for their thoughtful comments. We have addressed appropriate major changes, including addition of statistical analyses, quantitative measurements of Akkermansia, clarification on murine models, and revision of manuscript presentation to better reflect our original goals. While metagenomic sequencing to identify functional changes was outside of the manuscript original scope, we have addressed this limitation (and others) in the discussion. Specific point-by-point responses are included here. Please note that line numbers match the marked-up version of the manuscript.

Reviewer #2 (Comments for the Author):

The manuscript by Flores & Seekatz investigated the effect of carbohydrate complexity on the gut microbiota in a murine model. Using conventional mice and 16S rRNA gene sequencing, the researchers reported that low-complexity carbohydrate diets containing simple sugars resulted in significantly lower bacterial diversity, altered microbiota composition, and higher Akkermansia abundance compared with high-complexity diets. The study provides an important scientific foundation for understanding diet-microbiome interactions. While the manuscript is logical and meaningful, several methodological aspects require clarification or improvement to support the impact of the research to meet the publication criteria. The concerns are described in more detail below:

Major concerns

- The study's central limitation is its reliance on 16S sequencing, which provides bacterial compositional changes but offers no direct evidence of microbial functional outcomes. The following analyses will significantly enhance the impact of the work: (1) Metagenomic sequencing to identify shifts in functional gene families and metabolic pathways (e.g., GHs); and/or (2) Metabolomic analysis (e.g., by LC-MS or NMR) to quantify key microbial metabolites (e.g., SCFAs). (3) As a minimum alternative, PICRUSt2 could be used for functional prediction from the existing 16S data.

We agree with the reviewer that functional characterization via the referred methodologies is necessary to provide evidence of functional outcomes. Indeed, some of these (more involved) methods are ongoing, focused on integrating functional and metabolic consequences of the microbiome changes. That said, the current manuscript is focused on taxonomic redundancy to complement compositional microbiome analyses. While PICRUST2 can be used to infer functional prediction, this would not strengthen the main goal (compositional dynamics and taxonomic redundancy under the dietary experimental design) of the manuscript.

To emphasize this point, we have more specifically acknowledged the limitations of using 16S rRNA sequencing alone, (lines 616 – 618; 659 – 661; 686 – 697) in a new conclusion paragraph as suggested by reviewer #3). We appreciate the reviewer's suggestion and believe these future analyses will substantially extend the mechanistic understanding of the observed microbial shifts.

- The effect of diet on total bacterial load remains unmeasured. A qPCR analysis of total

bacteria (targeting the 16S gene) is recommended to determine if the observed shifts in relative abundance reflect true changes in bacterial density.

We thank the reviewer for the feedback. We agree that measuring total bacterial load using qPCR targeting the 16S rRNA gene could provide useful context for estimating total bacterial load and potentially aid absolute abundances. However, ours and others' previous endeavors in total bacterial loads based 16S rRNA quantification alone in mice do not substantially change unless large perturbations (i.e., antibiotic treatment) are implemented (PMID: 22198617). As such, 16S rRNA qPCR of total bacterial loads are unlikely to meaningfully change our current conclusions. To extend bacterial changes beyond relative abundance would require spike-ins of set standards prior to sequencing, which was not included in sample preparation and sequencing (PMID: 31195972, 31927795) or design unique qPCR targets per bacterial species of interest. Of note, standard spike-in methodologies are rarely implemented across microbiome studies despite their utility in improving accuracy of estimating bacterial trends over time. While certainly targeting bacterial species of interest (see below for Akkermansia) is relevant to the study, we feel that our current conclusions in this manuscript about compositional changes and taxonomic redundancy from dietary interventions are highly reproducible. This is demonstrated by 1) consistent longitudinal changes following the diet changes within individual mice and 2) similar trends across 2 different mouse cohorts (experiments conducted a year apart) and across cages / sexes, albeit with some variability, which is referenced in the manuscript. Finally, 16S rRNA qPCR to estimate total bacterial load would not aid resolution of the taxonomic redundancy changes we observe across these cohorts, which was a major goal of this study.

- Line 203, the authors claimed that "the rate of weight gain for mice fed LCD was significantly slower compared to those maintained on HCD (linear mixed-effects model, $p < 0.0001$)." However, the current Figure 1D does not fully support this conclusion. A spaghetti plot connecting the weight of the same mouse from Week 4 to Week 8 may be helpful to visually demonstrate the rate of weight gain.

We have added a spaghetti plot showing the trajectory of individual mice from Week 4 to Week 8 in a revised Supplementary Figure S1. Our initial analysis used a linear mixed-effects model with random intercepts only. Inclusion of both random intercepts and slopes for time suggests the difference in the rate of weight gain between the LCD and HCD groups shows trend toward significance ($p = 0.074$), rather than reaching statistical significance (manuscript lines 245 – 248; 498 – 502). Importantly, the overall pattern of reduced weight gain in the LCD group remains consistent with the visual trends observed in the spaghetti plot.

- Line 224: Could the authors further explain the rationale for using AMOVA? Microbial beta-diversity is widely analyzed by PERMANOVA/Adonis (https://pmc.ncbi.nlm.nih.gov/articles/PMC5695134), with both P-value and R-squared value reported in the main text and figures.

PERMANOVA, as implemented in the vegan R package (adonis), has now been used and is now referenced / added to figure legend. The results remain statistically significant and are consistent with the conclusions drawn from the original AMOVA analysis.

- Line 264: The choice of MaAsLin2 for differential abundance analysis should be justified. A study (https://www.nature.com/articles/s41467-022-28034-z#Abs1) showed that ALDEx2 and ANCOM-II produce the most consistent results across studies and agree best with the intersection of results from different approaches.

MaAsLin2 was chosen because it allows modeling of covariates and provides robust testing of associations between microbial taxa and experimental variables while maintaining statistical power. We recognize that more conservative methods, such as ALDEx2 and ANCOM-II, are often recommended for controlling false positives across studies. However, as noted in the cited study, MaAsLin2 has been shown to produce results more consistent than random expectation across multiple datasets, demonstrating reasonable reproducibility despite known technical and biological variability. To ensure the robustness of our results, we also performed complementary analyses using ALDEx2 and ANCOM-BC.

ALDEx2 identified 31 significant taxa (either MCD or LCD versus HCD), whereases ANCOM-BC, an updated evolution of the original ANCOM method with enhanced bias correction and modeling of compositional data, identified 272 significant data under the same comparisons. MaAsLin2 results fall between these extremes, providing a balance of statistical power and reliability. Importantly, despite differences in the number of significant taxa detected the major biological themes were consistent across all three methods:

- *Akkermansia was consistently more abundant in LCD versus HCD.*
- *Lachnospiraceae were generally more abundant in HCD compared to lower complexity diets.*

These observations indicate that the main signals reported in the manuscript are robust independent of the statistical choice. Based on these considerations, we believe MaAsLin2 provides an appropriate balance of power and reliability for our dataset. We have added text to Methods (lines 369 – 370) to clarify the rational for this choice and acknowledge the trade-offs relative to alternative differential abundance methods.

- Figure 3A: (1) The plot suggests a potential cage effect. The sex and cage effect should be tested and included as covariates in the statistical analysis of the microbiome data. (2) The use of multiple shades within a phylum is distracting. A single, distinct color per phylum is recommended for clarity.

We thank the reviewer for this important suggestion regarding potential cage and sex effects. In response, we have revised our microbiome statistical analysis to more appropriately account for these variables. Specifically, the model has been updated in

MaAsLin2 to include treatment group and sex as fixed effects and cage as a random effect. In addition, we have revised the normalization and modeling approach to use centered log-ratio (CLR) normalization, with no additional transformation and a linear model framework, replacing the previous Total Sum Scaling (TSS) normalization and log-transformed default approach. These updates provide a more robust framework for assessing differential abundance while accounting for potential confounding variables.

Following re-analysis, we observe some differences in specific differentially abundant taxa (i.e., certain ASVs such as ASV057 Clostridium sensu stricto are no longer significant under the updated model, while additional sex-associated differences are now detected). Importantly, the primary biological conclusions of the study remain unchanged, including the increase in Akkermansia in mice fed lower complexity diets, and decreases in ASVs belonging to Lachnospiraceae. All relevant text, figures, and statistical results have been updated accordingly.

Regarding point (2), the color scheme in Figure 3A groups genera by their associated phylum using shades of the same color to highlight phylogenetic relationships. The figure legend has been updated to clearly describe this scheme for improved readability.

- Figure 3B: The CLR (centered log-ratio) abundance is recommended to represent relative abundance in visualizing and analyzing compositional data (https://pmc.ncbi.nlm.nih.gov/articles/PMC5695134).

We thank the reviewer for the suggestion regarding the appropriate representation of compositional microbiome data. We have updated Figure 3B to use centered log-ratio (CLR) transformed abundance rather than log2(relative abundance + 1). This change aligns the visualization with the updated statistical framework used in our revised differential abundance analyses. We have updated the figure and its legend to reflect this transformation and to clearly indicate how values are presented.

- Figure 3C: The figure and its legend require a more precise description. Based on the legend title, my interpretation is that the node size shows ASV count within each taxa, while the color (black vs brown) represents the log2 fold-change to indicate the taxa is more or less abundant. Additionally, a standard diverging color scheme (e.g., blue for decrease, red for increase) would be more intuitive than black/brown.

We thank the reviewer for the feedback. We have clarified the legend for Figure 3C to more clearly describe how node size reflects ASV counts within each taxon and how color represent the log2 fold-change in abundance. We have also replaced the original black/brown color scheme with a standard diverging palette (blue for decrease and red for increase) to improve clarity.

Minor concerns

- To validate the key finding on Akkermansia, qPCR analysis targeting this species is recommended. This is necessary to confirm whether its increased relative abundance

represents an absolute increase in its population or is a compositional effect driven by the decrease of other taxa.

We thank the reviewer for the suggestion. In response, we have performed qPCR analyses targeting Akkermansia in mouse cecal samples to directly address this point, demonstrating increased Akkermansia abundance in mice fed LCD, concurring with our 16S rRNA results (Figure 3E). Materials and Methods and Results have been revised accordingly (lines: 325 – 347; 470 – 474).

- Line 290: Since the author used the number of unique ASVs in the redundancy analysis, it will be more reasonable and consistent to use the index "observed ASVs" to represent alpha diversity and move the current Figure 2A to the supplement.

We thank the reviewer for this suggestion. While we acknowledge that observed ASVs provides a direct measure of richness, our study focuses on taxonomic redundancy, where both richness and evenness are important for ecological interpretation. For this reason, we present inverse Simpson diversity in the main figure, as it captures both richness and evenness, offering a more comprehensive reflection of changes in community structure across conditions.

We have highlighted that additional alpha diversity metrics, including observed ASVs (Sobs), Shannon, Simpson, and total sequence counts (Abundance), are provided in Supplementary Figure S3. Independent of what diversity metric was used, mice on LCD demonstrated decreased diversity.

Figure 1B: The mouse schematic is inaccurate and misleading, as the tail is incorrectly depicted and resembles that of a squirrel.

This has been amended.

- Figure 2 A, B, C, E: P values need to be added to clearly indicate the significance of the comparisons shown.

The corresponding p-values have now been added to the relevant panels in Figures 2 A, B, C, and E. They are also indicated in the figure legends to clearly denote the statistical significance of the comparisons shown.

In summary, this study reveals diet-induced alterations in bacterial diversity, taxonomic redundancy, and specific species under distinct carbohydrate complexity. However, the conclusions are currently limited to taxonomic observations. It is recommended to address the major points concerning functional validation, statistical rigor, and data presentation. Incorporating these changes will significantly enhance the manuscript's contribution to the field.

We agree with and thank the reviewer—our goal in this study was indeed limited to taxonomic observations, specifically focused on microbiota compositional changes and

taxonomic redundancy in response to the designed diets. While our discussion brings up specific reasons for these changes (which would require functional analyses to validate), our manuscript does not over-emphasize conclusions beyond what we analyzed, i.e. taxonomic changes. Our discussion on functional interpretations from these data remains appropriate to what is known about specific bacteria and their functions (example: mucin degradation by Akkermansia), within the context of limitations to our study. We hope that the above responses and revised discussion points / analyses now strengthen these conclusions.

Reviewer #3 (Comments for the Author):

The study from Flores and Seekatz investigates how gut microbial community in murine gut reacts to different carbohydrate sources that differ in complexity of their chemical structure. The notion appears to arise from broader claims suggesting that human-driven environmental changes lead to evolutionary simplification of ecosystems. Although such patterns were shown in other ecological systems, the application of this concept to human evolution and dietary transitions is far less straightforward. Urbanization, in the context of diet, does not really produce simplified dietary environment, but rather complex. The availability of variety of foods, transportation, and having access to different food types contribute to the complexity. Supporting this, the study from DeFillipo et al 2010 showed that urban European children exhibited more complex gut microbial composition than children living in in Burkina Faso, who consume traditional and fiber-rich diets https://pubmed.ncbi.nlm.nih.gov/20679230. Given this, the way intro, abstract, and importance sections were currently written makes it very hard to grasp the hypothesis and objectives of the study. The ultimate goals are about the effects of nutrient complexity and availability on microbial diversity. The narrative sets the readers up to expect an investigation grounded in evolutionary ecology rather than a mechanistic test of how nutrient availability and CHO structural complexity shape microbial community. Importantly, this focus does not emerge until line 80. To improve clarity, the authors should reframe the introduction from the outset, reflecting the true drivers of the experimental design and results.

We thank the reviewer for this thoughtful comment and have improved the introduction to more clearly communicate the primary hypothesis and objectives of the study. Our intention was to place the work within a broader ecological context in which changes in dietary inputs can influence microbial community structure and functional potential. However, we agree that the current text may delay the introduction of the central experimental question – how does carbohydrate diversity and complexity shape gut microbial community composition, diversity, and taxonomic redundancy.

We have revised Introduction, Abstract, and Importance sections to more directly introduce the mechanistic focus of the study earlier in the text. Specifically, the revised Introduction now foregrounds the role of carbohydrate structural diversity and nutrient availability as key drivers of microbial community assembly in the gut.

Regarding the example raised by the reviewer, the study by DeFilipo et al 2010 indeed highlight differences in gut microbiota between Burkina Faso children consuming high-fiber, ancestral-type diets and European Union children consuming Western diets, reporting higher microbial richness and diversity in children from Burkina Faso compared with children from Europe. These findings support the broader concept that dietary composition and fiber intake influence microbial diversity and metabolic output, including short-chain fatty acid production. To improve clarity and context, we have incorporated additional references addressing how carbohydrate complexity and availability shape gut microbial ecology.

In addition to concerns about narrative, the study design is questionable and raises concerns over the interpretation of the results. Particularly, Figure 1A is very confusing. Maltodextrin 10 is not labeled as complex carbs in the figure, which is incorrect as maltodextrin is a polysaccharide. In addition, LCD contains 12.5% complex CHO but there is no yellow label. This seems like an error and adds to the confusion on what authors consider complex CHO. Furthermore, labeling fiber differently than CHO is scientifically inaccurate. The figure seems to treat cellulose as the only fiber source, which is reductive and inaccurate. Even though cellulose is not nutritionally valuable from host perspective, some gut microbes utilize cellulose https://pmc.ncbi.nlm.nih.gov/articles/PMC7615765/. Additionally, certain carbohydrate sources labeled here as complex carbs such as corn starch can function as resistant starch, which is operationally a form of dietary fiber. Without correcting these foundational ambiguities, the mechanistic conclusions about carbohydrate complexity and microbial diversity remain difficult to evaluate.

We thank the reviewer for the feedback. First, we acknowledge the error in the labeling of maltodextrin 10 in the original figure. Maltodextrin is a polysaccharide and was included in our calculations as a complex carbohydrate source; however, it was incorrectly highlighted as a simple carbohydrate source in Figure 1A. This has now been corrected in the revised figure.

More broadly, we recognize that the figure did not clearly convey the intended experimental design. The custom diets were formulated based on a modified standard purified rodent diet from Research Diets (D11112225, Table S1), which served as the nutritional template for macronutrient composition and non-carbohydrate ingredients. From this baseline formulation, we modified the composition of digestible carbohydrate sources. The goal of this study was to generate a gradient in the diversity and polymer complexity of digestible carbohydrate substrates, rather than to contrast carbohydrates with dietary fiber. To accomplish this, all diets were formulated to be isocaloric and matched for macronutrient composition, with a defined amount of cellulose included across diets to maintain consistent fiber content. This approach allowed us to minimize variation in total dietary fiber, which is known to strongly influence the gut microbiome, and instead focus on how differences in the diversity and structural complexity of carbohydrate substrates influence microbial community composition.

Specifically, the control diet (HCD) contained a mixture of starches (corn, wheat and potato) and simple sugars, the intermediate diet (MCD) contained a reduced diversity of carbohydrate sources (corn starch and simple sugars), and the low-complexity diet (LCD) contained only simple sugars. Maltodextrin 10 was included at the same level in all diets as required for pelleting and therefore did not represent a variable treatment component.

We agree with the reviewer that cellulose represents only one form of dietary fiber and that some starch sources can contain resistant fractions that function as fermentable substrates for gut microbes. In our formulation, however, cellulose was used as a defined fiber source to maintain comparable fiber levels across diets, allowing the primary experimental variable to remain the diversity and complexity of digestible carbohydrate substrates.

We have added to Materials and Methods lines 249 – 274 to more clearly describe the classification of carbohydrate sources and rational underlying the diet design.

In addition to the issues with diet categorization, animal study design raises major concerns, particularly regarding microbial variability across animals and cages. The mice used in the study ranged from 6 to 10 weeks of age, which represents a substantial developmental window from a microbiome perspective. Is there preliminary data showing the gut microbiome is stable across time without any perturbations? This age range may also explain the sex and cage variation mentioned in lines 252-255. If animals entered the study with differing microbiome maturity levels, these pre existing differences would likely amplify cage effects. Another inconsistency is found in the labeling and interpretation of Experiment 2nd in Figure 3, where the text indicates the variation observed in these cages prompted authors to conduct the 2nd validation experiments.

We agree with the reviewer in that cage variation (i.e., 'cage effects') is a known factor in murine studies. Both in our hands (PMIDs: 26169276, 25547352, 40454811, 33844988) and others' (40126017, 33531483, 40689613), some amount of cage effects are unavoidable when conducting mouse studies. As such, our animal study design purposefully included multiple cage variants and sexes, detailed below for each aspect brought up by the reviewer:

1) *6-10 weeks in mice is a standard age window to conduct begin murine studies, representing young / young-adult age stage of mice. This is unlikely to explain cage effects or variability within a breeding colony, as mice are typically weaned from their mothers at 3-4 weeks, at which point litters are split into individual cages and are in contact with each other for > 2 weeks, a standard time window for 'microbiome stabilization'. Many labs (including ours) order mice from external vendors, which may ship all female vs male mice in a larger boxes containing more than the standard <5 mice per cage. Any mice received by our facility are thus likely to be exposed to each other (within each sex group) during shipment. While we do not have complete control to dictate that all mice come from the*

same breeding colony / barrier, upon receipt, we try to order from the same barrier across experiments. We also randomly assign mice upon receipt into cages of 3 each to purposefully avoid cage effects. Despite this, we feel inclusion of cage representatives is a strength of our study (example: each diet group consisted of 6 cages of 3 mice each, 2 sexes).

2) *Sex has been demonstrated to impact cage effects and sometimes experimental outcomes. This is why we purposefully included 3 cages of male and female mice each per treatment.*
3) *What is known to influence cage effects are breeding colonies and diet (PMIDs: 40126017, 30911125). While we try to request from the same breeding colony / barrier from the vendor each time, this is not guaranteed, nor can we control microbiome drift over time from the same barrier. Furthermore, diet changes across facilities are also known to influence the microbiome, which can also sometimes influence experimental outcomes. This is why we placed all received mice on the same diet upon receipt for 4 weeks to allow substantial microbiome stabilization. While we still cannot control for any initial cage effects that may have been present, our data (see Bray-curtis longitudinal results in Figure 2E) suggests that mice adapt to the diet < 2 weeks.*

Furthermore, in the microbiome analysis, the authors combine the fecal sample 16S data with the cecal sample which is represented in Figure 2C. 1) Compositionally and naturally, fecal samples which are derived from distal colon and cecal samples are distinct. Naturally, cecal samples will also contain microorganisms that are also attached to the mucus whereas fecal samples contain flushed out microorganisms, aka ileal content. Therefore, combining these data in the same ordination or diversity analysis without accounting for inherent biological variance introduces confounding interpretation. 2) Another drawback is that the ambiguity on what counts as technical replicates and what counts as biological replicates. A fecal sample collected from mice housed in the same cage should be treated as technical replicates, not biological replicates because mouse co-housed in the same cage will share a common microbial environment and should naturally converge toward similar microbial profiles. Thus, a cage constitutes an n=1 for microbiome analysis unless individually housed. Without explicit sample size reporting, the validity of statistical comparisons cannot be assessed. 3) Additionally, it is unclear whether the cecal samples included in Figure 2 and subsequent analyses were collected immediately after the gut transit/permeability assays or whether they derive from a separate cohort of mice. If these samples were obtained following transit or permeability measurements, the physiological perturbations imposed by those assays could independently alter the cecal microbiota. This ambiguity significantly undermine confidence in the microbiome analyses and limit the reliability of the conclusions drawn.

We agree that fecal and cecal samples represent related but distinct biological compartments, and that direct comparison should be interpreted with this distinction in mind. In the original figure, fecal and cecal samples were both included in the ordination to visualize the overall pattern of community variation; this was intended as a descriptive comparison rather than a statistical test of equivalence between sample

types. To address this concern, we note that statistical analyses were performed separately for fecal and cecal samples (originally using an AMOVA) and indicated as significant. For clarity and consistency with current best practices, these analyses have now been updated to PERMANOVA and are reported accordingly in the revised figure legend and main text. Both sample types show significant effects of diet on community structure, and the overall pattern of separation by treatment is consistent across fecal and cecal samples.

Each sequenced sample represents one biological replicate (one mouse), with fecal samples collected longitudinally from that individual and once cecal sample collected at termination. Longitudinal samples from the same mouse are not treated as independent biological replicates for cross-sectional analyses but are used in longitudinal modeling where appropriate. At each timepoint, a single fecal sample per mouse was analyzed, unless a mouse did not void naturally on a given day, in which case no sample was collected. Thus, for cross-sectional comparisons, each mouse contributes one biological replicate per sample type. We acknowledge that mice housed in the same cage share a common environment and that co-housing can influence the microbiome through coprophagy and shared microenvironmental exposures. However, it is standard practice to treat individual animals as biological replicates if they are from separate cages or experimental units. While microbiota typically follow cage effects, experimental outcomes in more disease-like models (such as death or adverse signs of disease) typically do not. As such, we adhere to standard practice of treating individual mice as individual points when conducting mouse studies. Whenever possible, we co-house mice as they are social animals and individual housing can induce stress to the animal, which has been shown to alter the gut microbiome (PMIDs: 12689611, 25028050, 27633986). Importantly, our study design purposefully used multiple cages / experimental replicates to offset cage effects. While certainly this adds variability, we observed consistent results across all cages / experiments. We have now included a statement in Methods clarifying how biological replicates are defined in this study and how co-housing was accounted for in the analysis (lines 245 – 248; 353 – 356). Individual mouse metadata is also available under Table S2.

We clarify that cecal samples included in Figure 2 and subsequent analyses were collected from the same cohort of mice used in the first experimental replicate. Cecal sample collection occurred upon euthanasia at the end of the 8-week study period following a brief recovery period after the procedures pertaining to sample collection for intestinal permeability analysis. Fecal samples were collected prior to the physiological assays and at scheduled intervals following those procedures to minimize confounding effects. Based on the longitudinal fecal data, we did not observe abrupt or systematic shifts in community composition associated with the timing of these assays, and diet effects remained the primary drivers of microbiome differences.

Another concern relates to the caloric intake analysis, which directly impacts the interpretation of the dietary intervention. The authors report that female mice consumed fewer calories overall. This finding challenges the assumption that carbohydrate intake remained equal across groups, because actual intake, not the theoretical diet

composition, determines nutrient exposure. If female mice consumed fewer total calories, then their absolute carbohydrate intake was not held constant, which could independently influence microbial composition and physiological outcomes. This discrepancy could also contribute to variability observed across cohorts and sex. These were never addressed as limitations in the discussion. Moreover, the validation of this effect is insufficient, as the authors do not correlate caloric intake with microbial outcomes or examine whether intake differences could account for the reported diversity shifts. If cage is the true biological unit for dietary exposure, then using individual mice as independent microbiome replicates creates a mismatch between the exposure variable (intake) and the microbiome outcome.

Food consumption was measured at the cage level, which is standard practice in group-housed rodent studies. Total caloric intake per cage was averaged across the three mice housed in each cage to estimate per-mouse intake. As noted by the reviewer, female mice consumed fewer calories overall than males; however, within each sex there was no significant differences in caloric intake between dietary treatment groups. Because the primary experimental comparison in this study is between diet compositions rather than between sexes this result indicates that dietary treatments did not differentially alter caloric intake within sex, and therefore differences in microbial composition are unlikely to be explained by treatment-driven differences in energy consumption.

To further clarify this point, we have now expanded the description of caloric intake analyses in Methods and Results sections (lines 238 – 244) and added a brief statement in Discussion (lines 648 – 649) acknowledging that food intake was measured at the cage level and therefore individual variation in intake among co-housed mice cannot be completely excluded. Because intake did not differ significantly across diet groups within sex, total caloric consumption is unlikely to confound interpretation of diet-depended microbiome shifts. We have included additional multivariate analysis of microbiome composition including sex as a factor in a differential analyses (see responses to Reviewer #2; lines 246 – 248 and throughout manuscript). The analysis confirms that diet remains a significant driver of community structure, with diet explaining a larger proportion of variance than sex. These results further support the interpretation that the observed microbial differences primarily reflect dietary carbohydrate composition rather than differences in caloric intake.

Once again regarding the experimental unit, mice were housed three per cage with multiple cages per sex and diet treatment, providing balanced cage representation across experimental groups. Individual mice were treated as biological replicates for microbiome analyses because each mouse represents an independent host-associated microbial community, a convention commonly used in murine microbiome studies when cages are balanced across treatments (lines 353 – 356).

Taken together, these clarifications indicate that while sex-related differences in overall caloric intake were observed, dietary treatment did not differentially affect caloric intake

within sex, and additional multivariate analyses confirm that diet remains the dominant factor associated with microbiome variation in this study.

Minor revisions:
Line 242, cited wrong figure.

This has been amended.

Figure 2 indicates the authors performed AMOVA, do they mean PERMANOVA?

Thank you for the feedback. As described in our response to Reviewer 2, the analysis has been updated to PERMANOVA in the revised manuscript.

Because in the M&M section, they mention that they performed PERMANOVA.

Yes, see above.

Figure 3A cages 907, 9098, 911 and 912 contain a green color that is not depicted in the legend.

The legend has been corrected in the revised figure.

From Figure 3A, it is very hard to understand the genus levels as authors claim in lines 256-261 because the figure legend only indicates phyla level. The authors should cite Table S2 at the same time.

Thank you for the feedback, The appropriate supplementary table citation has now been added to the revised manuscript.

Lines 297-299, percentages should be given.

From our end, percentages across the groups were already reported (lines 448 – 450 in the revised marked up manuscript).

Line 193 in the Results section, the "host health" is an overinterpretation of the results although the measurements include calories, body weight, and fat mass there is no metabolic health measurements in serum.

We agree. To avoid overinterpreting the data, we have updated the section title from “host health” to “host physiology”. This change more accurately reflects the metrics we measured (body weight, fat mass, transit time etc.) and avoids implying systemic metabolic health outcomes, which were not directly assessed in the study.

There is no clear conclusion paragraph.

A conclusion paragraph has now been added to the revised manuscript (lines 686 – 697).

Line 350, the discussion text was submitted without proof reading, still include draft version. The authors' note to themselves: (ref)

The discussion has been carefully proofread, and the draft note has been removed in the revised manuscript.

Lines 375-376, the Akkermansia abundance in LCD is repeated twice.

This has been rephrased.

Line 334 needs to mention unclassified.

The text has been updated to include “unclassified” (line 516 in marked up manuscript).

Line 182 data visualization is a repeat in the same sentence.

This has been amended (line 559 in marked up version).

Re: Spectrum03107-25R1 (**Decreased carbohydrate sources reduce microbial diversity and taxonomic redundancy in the murine gut**)

Dear Dr. Anna Maria Seekatz:

Your manuscript has been accepted, and I am forwarding it to the ASM production staff for publication. Your paper will first be checked to make sure all elements meet the technical requirements. ASM staff will contact you if anything needs to be revised before copyediting and production can begin. Otherwise, you will be notified when your proofs are ready to be viewed.

Data Availability: ASM policy requires that data be available to the public upon online posting of the article, so please verify all links to sequence records, if present, and make sure that each number retrieves the full record of the data. If a new accession number is not linked or a link is broken, provide production staff with the correct URL for the record. If the accession numbers for new data are not publicly accessible before the expected online posting of the article, publication may be delayed; please contact ASM production staff immediately with the expected release date.

Publication Fees: For information on publication fees and which article types have charges, please visit our website. We have partnered with Copyright Clearance Center (CCC) to collect author charges. If fees apply to your paper, you will receive a message from no-reply@copyright.com with further instructions. For questions related to paying charges through RightsLink, please contact CCC at ASM_Support@copyright.com or toll free at +1-877-622-5543. CCC makes every attempt to respond to all emails within 24 hours.

ASM Membership: Corresponding authors may join or renew ASM membership to obtain discounts on publication fees. Need to upgrade your membership level? Please contact Customer Service at Service@asmusa.org.

Embargo Policy: A press release may be issued as soon as the manuscript is posted on the Spectrum Latest Articles webpage. The corresponding author will receive an email with the subject line "ASM Journals Author Services Notification" when the article is available online.

The ASM Journals program strives for constant improvement in our submission and publication process. Please tell us how we can improve your experience by taking this quick Author Survey.

Thank you for submitting your paper to Spectrum.

Sincerely,
Steven Frese
Editor
Microbiology Spectrum
