# Supplementary material for: Decreased carbohydrate sources reduce microbial diversity and taxonomic redundancy in the murine gut
Source: Microbiol Spectr. 2026 Jul 2;14(8):e03107-25. doi: 10.1128/spectrum.03107-25 (PMC13436403; doi:10.1128/spectrum.03107-25)
Supplement: Supplementary file 2 [file spectrum.03107-25-s0001.pdf]

Supplementary Figures for

Reduced carbohydrate complexity alters gut microbial structure independent of total carbohydrate intake

Authors: Clara Flores[a] and Anna M. Seekatz[a*]

[a]*Department of Biological Sciences, Clemson University, Clemson, SC 29634, USA*

[*]Corresponding author

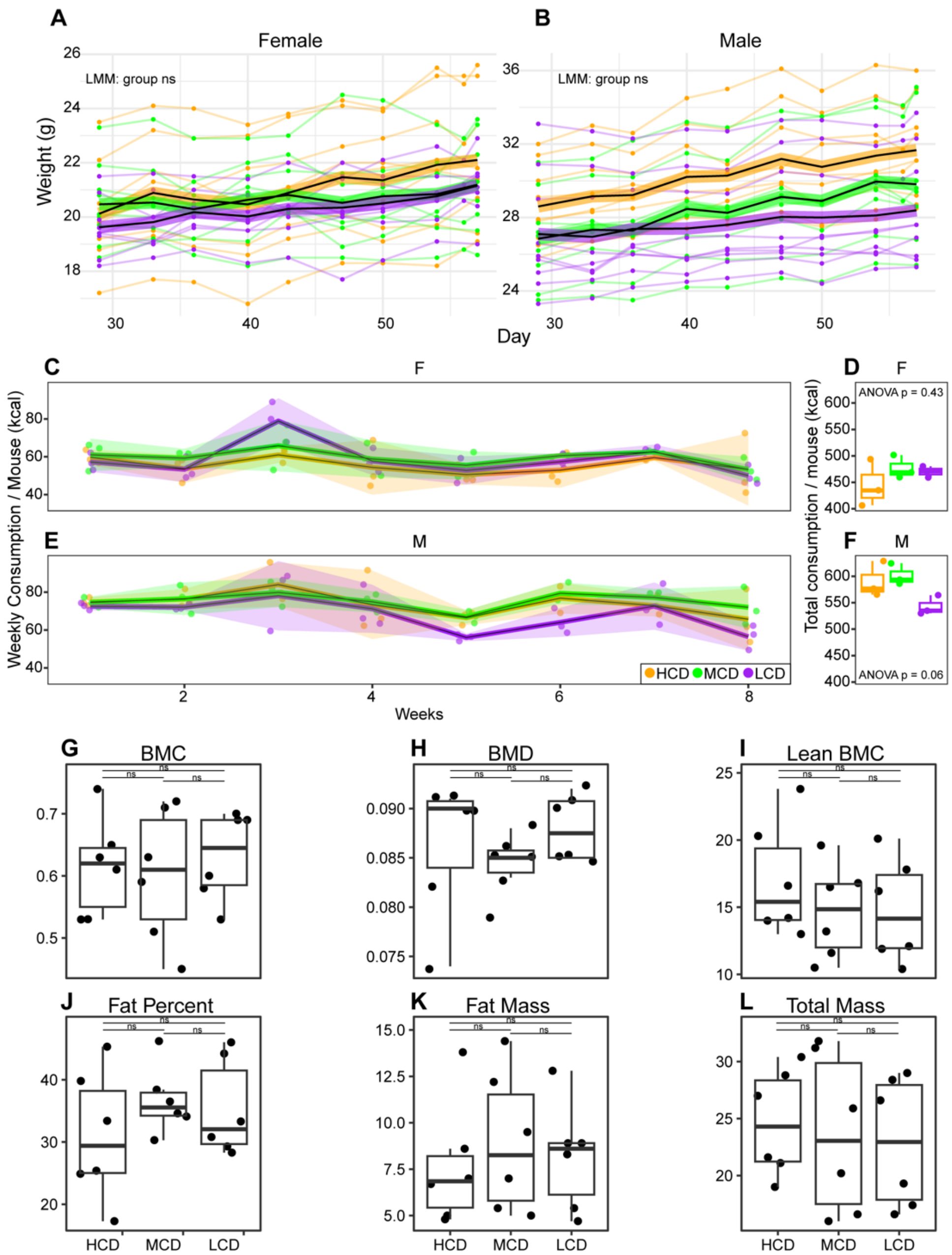

Figure S1. Dietary carbohydrate complexity did not significantly affect physiological parameters in mice. A-B) Spaghetti plot of longitudinal weight gain for **A)** female and **B)** male mice from diet switch to end of study. Longitudinal weight data were analyzed using a linear mixed-effects model with fixed effects for group, day, and their interaction, and random effects for specimen. Weekly **C)** and cumulative **D)** caloric intake (kcal) in female mice. Weekly **E)** and cumulative **F)** caloric intake (kcal) in male mice. Statistical analysis was performed using

ANOVA. **G-L)** DEXA scan-derived physiological measurements: **G)** bone mineral content (BMC), **H)** bone mineral density (BMD), **I)** lean BMC, **J)** body fat percentage, **K)** fat mass, and **L)** total body mass. Statistical analysis was performed using Kruskal-Wallis. ns, not significant.

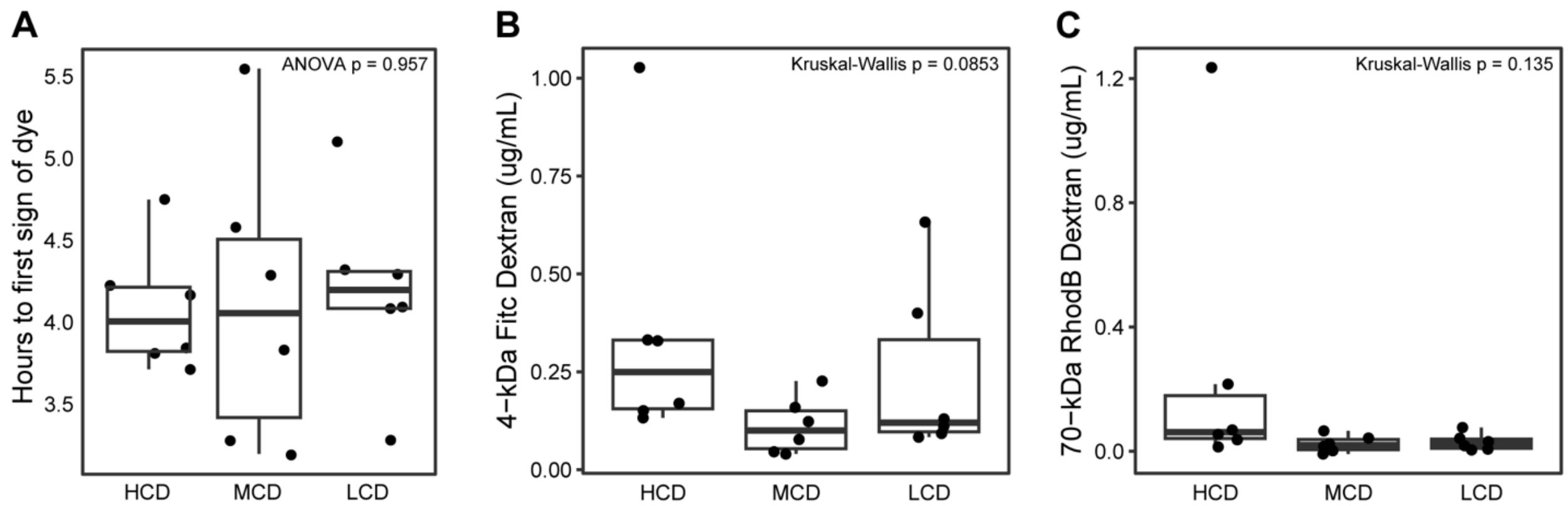

Figure S2. Dietary carbohydrate complexity did not significantly affect intestinal permeability. A) Gastrointestinal transit time (ANOVA, p = 0.957) and **B)** intestinal permeability measured by 4-kDa FITC-dextran and **C)** 70-kDa Rhodamine B concentrations in serum (Kruskal–Wallis test, p = 0.085 and p = 0.135, respectively).

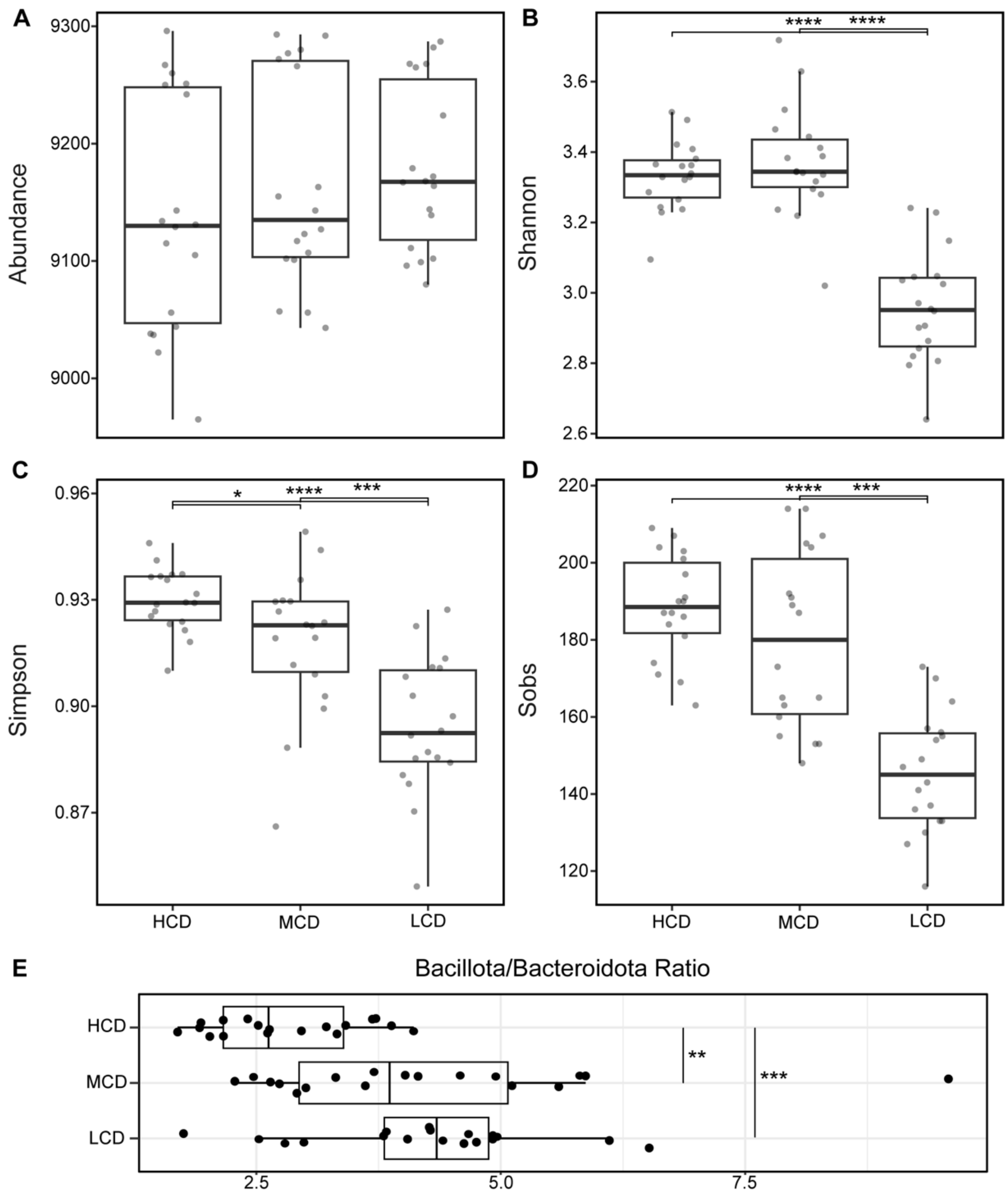

Figure S3. Lower complexity diets reduce gut microbiota diversity. Alpha diversity indices based on ASVs: **A)** Abundance **B)** Shannon index **C)** Simpson index and **D)** Observed sequences (Sobs). **E)** Bacillota/Bacteroidota ratio across diet group. Statistical analysis was performed using Kruskal-Wallis tests followed by pairwise Wilcoxon rank-sum tests (*p<0.05, **p<0.001, ***p<0.0001).

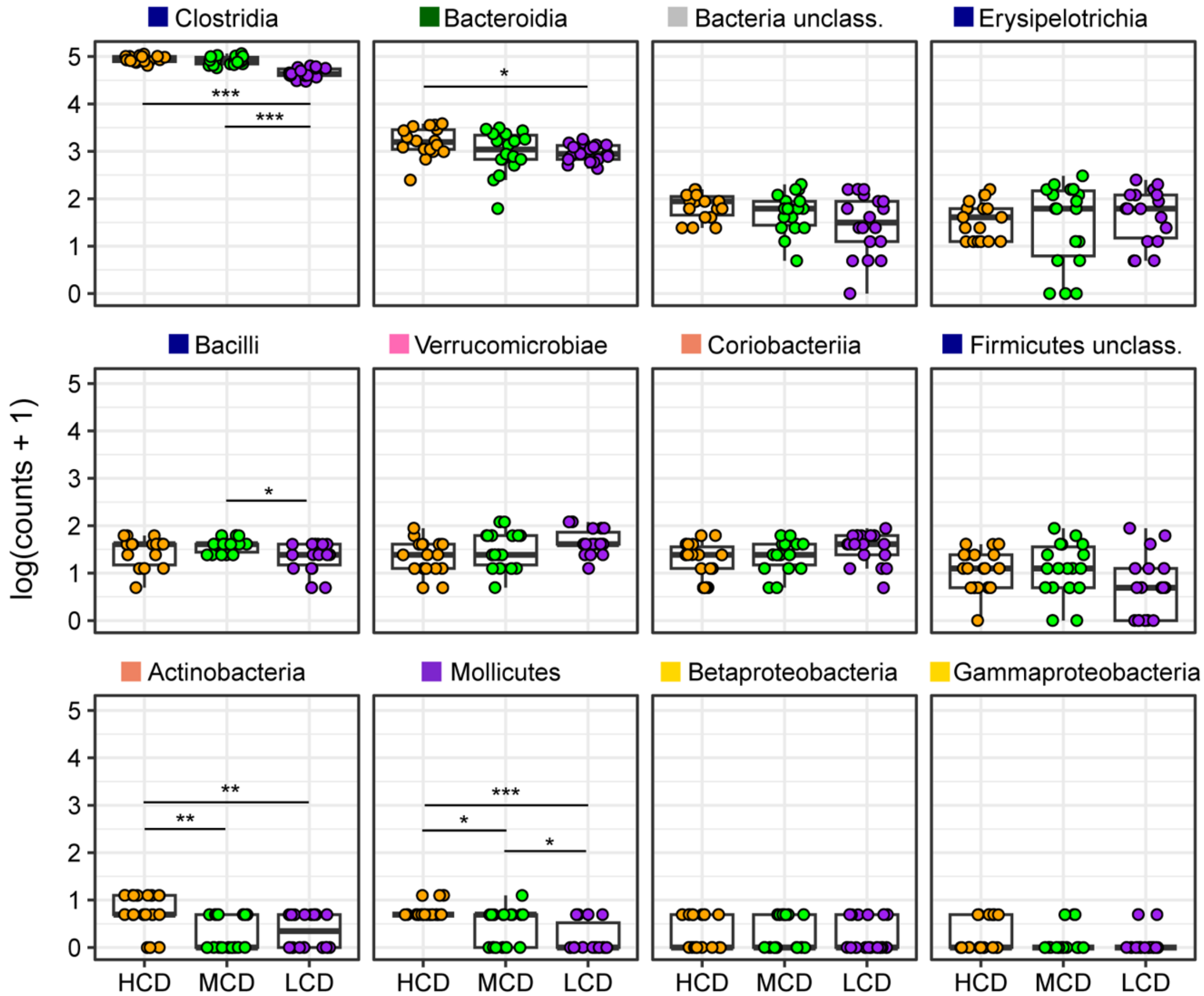

Figure S4. Taxonomic redundancy at the class level. Log-transformed (ln[count + 1]) unique ASV counts per mouse at the class level. Statistical comparisons performed using Kruskal-Wallis test with post hoc pairwise Wilcoxon rank-sum tests (*p<0.05, **p<0.001, ***p<0.0001).

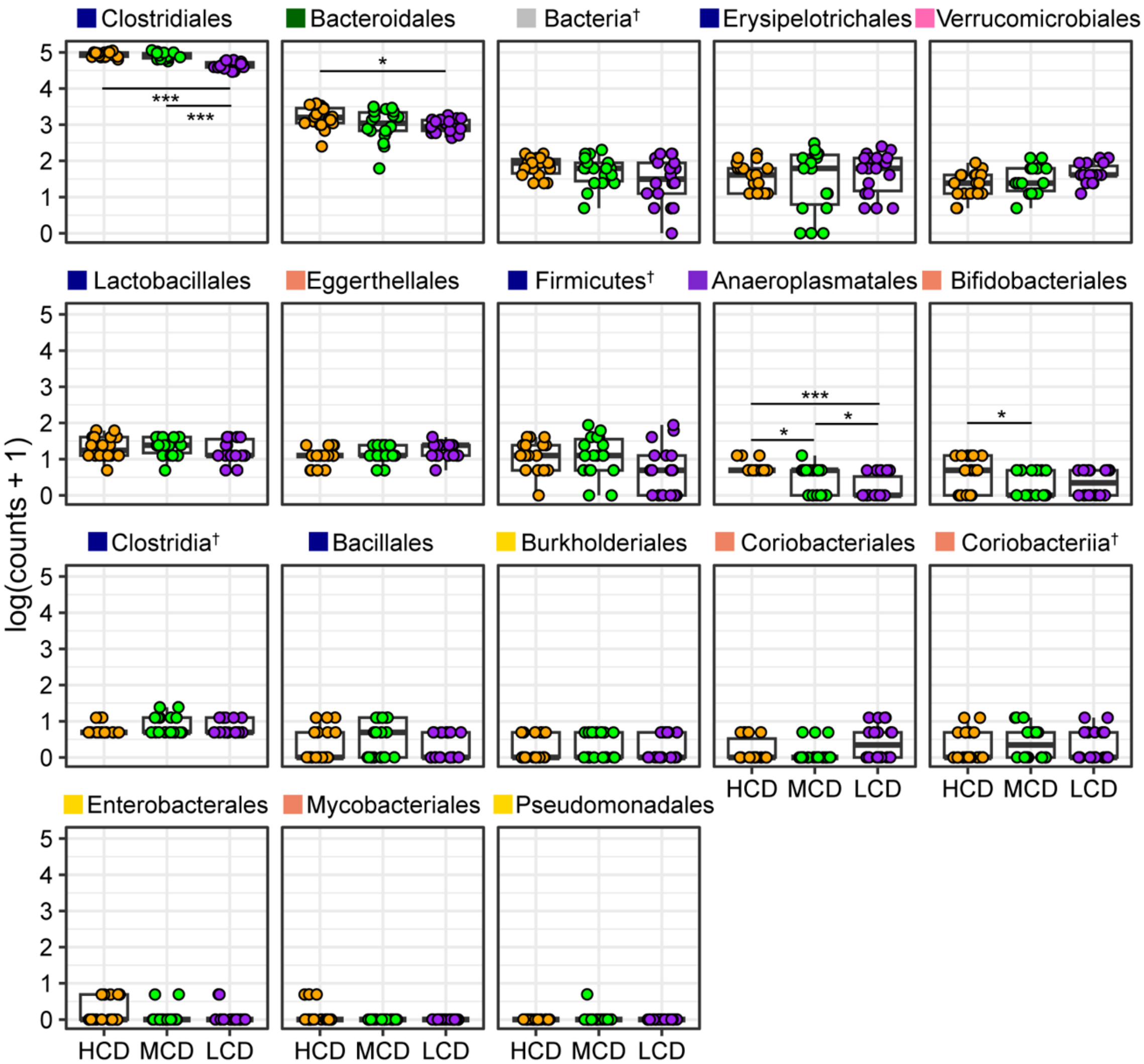

Figure S5. Taxonomic redundancy at the order level. Log-transformed (ln[count + 1]) unique ASV counts per mouse at the order level. Statistical comparisons performed using Kruskal-Wallis test with post hoc pairwise Wilcoxon rank-sum tests (*p<0.05, **p<0.001, ***p<0.0001).

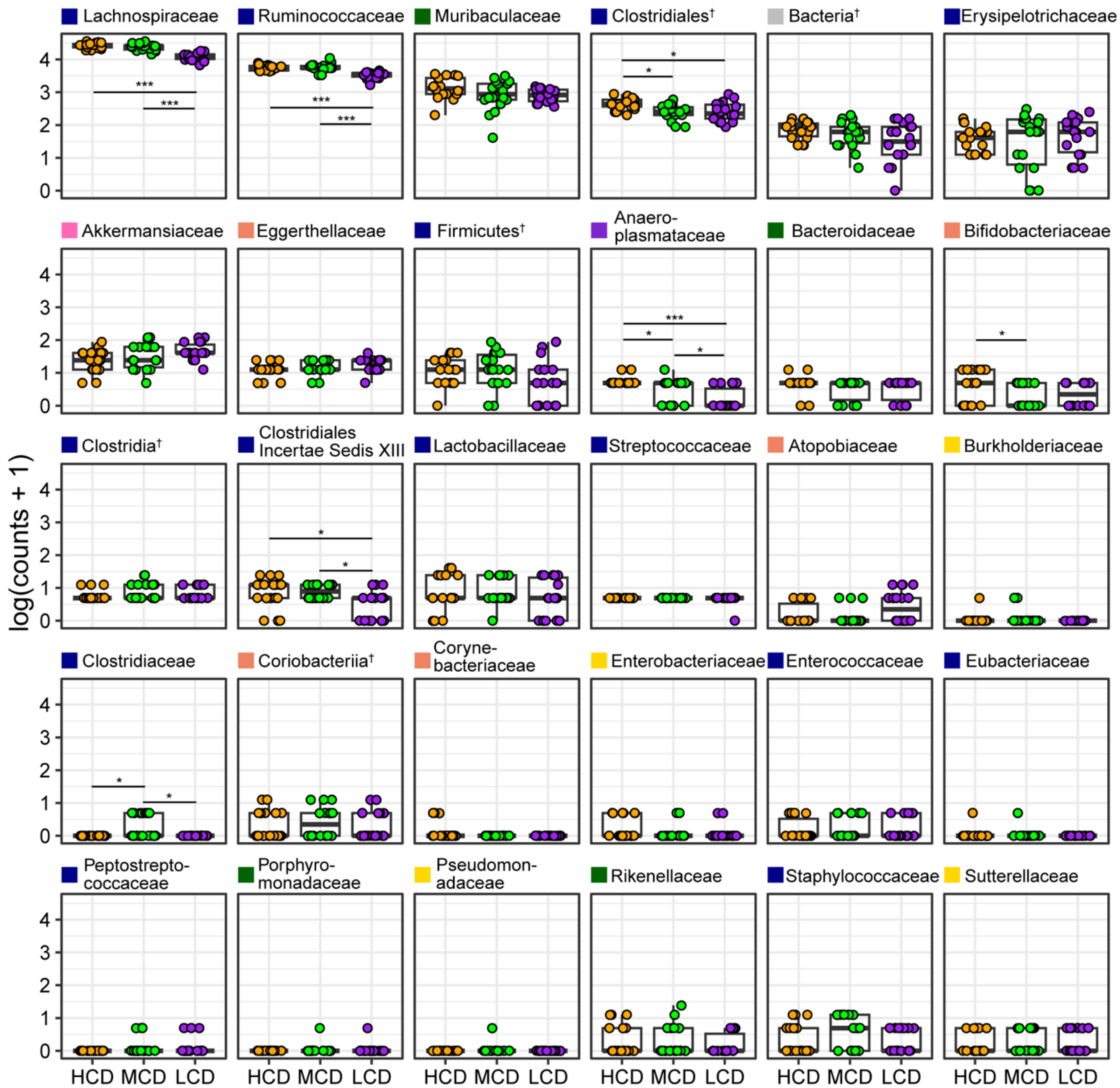

Figure S6. Taxonomic redundancy at the family level. Log-transformed (ln[count + 1]) unique ASV counts per mouse at the family level. Statistical comparisons performed using Kruskal-Wallis test with post hoc pairwise Wilcoxon rank-sum tests (*p<0.05, **p<0.001, ***p<0.0001).

SUPPLEMENTARY TABLES

Table S1. Diet breakdown. Summary of customized diets compared to Research Diets Open Standard Control (D11112225), High-complexity Diet (HCD, D22021105), Mid-complexity Diet (MCD, D22021106), and Low-complexity Diet (LCD, D22021107).

Table S2. Overview of project metadata. Summary of metadata associated with the project, including sample identifiers, and experimental conditions.

Table S3. Pairwise statistical comparisons of taxonomic redundancy at the genus level across diet groups. Summary of Kruskal-Wallis tests followed by pairwise Wilcoxon rank-sum tests on $\log_2(\text{count} + 1)$ unique ASV counts, averaged per diet group for each genus in the dataset, as shown in Figure 4. Significance levels are indicated as *p<0.05, **p<0.001, and ***p<0.0001.
